# Hypoxia-activated prodrug and antiangiogenic therapies cooperatively treat pancreatic cancer but elicit immunosuppressive G-MDSC infiltration

**DOI:** 10.1172/jci.insight.169150

**Published:** 2024-01-09

**Authors:** Arthur Liu, Seth T. Gammon, Federica Pisaneschi, Akash Boda, Casey R. Ager, David Piwnica-Worms, David S. Hong, Michael A. Curran

**Affiliations:** 1 The University of Texas MD Anderson UTHealth Graduate School of Biomedical Sciences, Immunology program, Houston, Texas, USA.; 2 The University of Texas MD Anderson Cancer Center, Department of Immunology, Houston, Texas, USA.; 3 The University of Texas MD Anderson Cancer Center, Division of Diagnostic Imaging, Department of Cancer Systems Imaging, Houston, Texas, USA.; 4 Mayo Clinic, Department of Immunology, Scottsdale, Arizona, USA.; 5 The University of Texas MD Anderson Cancer Center, Department of Investigational Cancer Therapeutics, Houston, Texas, USA.

**Keywords:** Angiogenesis, Therapeutics, Cancer immunotherapy, Hypoxia, Neutrophils

## Abstract

We previously showed that ablation of tumor hypoxia can sensitize tumors to immune checkpoint blockade (ICB). Here, we used a *Kras^+/G12D^ TP53^+/R172H^ Pdx1-Cre*–derived (KPC-derived) model of pancreatic adenocarcinoma to examine the tumor response and adaptive resistance mechanisms involved in response to 2 established methods of hypoxia-reducing therapy: the hypoxia-activated prodrug TH-302 and vascular endothelial growth factor receptor 2 (VEGFR-2) blockade. The combination of both modalities normalized tumor vasculature, increased DNA damage and cell death, and delayed tumor growth. In contrast with prior cancer models, the combination did not alleviate overall tissue hypoxia or sensitize these KPC tumors to ICB therapy despite qualitative improvements to the CD8^+^ T cell response. Bulk tumor RNA sequencing, flow cytometry, and adoptive myeloid cell transfer suggested that treated tumor cells increased their capacity to recruit granulocytic myeloid-derived suppressor cells (G-MDSCs) through CCL9 secretion. Blockade of the CCL9/CCR1 axis could limit G-MDSC migration, and depletion of Ly6G-positive cells could sensitize tumors to the combination of TH-302, anti–VEGFR-2, and ICB. Together, these data suggest that pancreatic tumors modulate G-MDSC migration as an adaptive response to vascular normalization and that these immunosuppressive myeloid cells act in a setting of persistent hypoxia to maintain adaptive immune resistance.

## Abstract 2

Vascular normalization therapy extends pancreatic cancer survival but evokes G-MDSC infiltration that subsequently blocks immune expansion.

## Introduction

Most patients with pancreatic ductal adenocarcinoma (PDAC) do not derive durable responses from immune checkpoint blockade (ICB) therapy (1, 2). Unlike ICB-responsive cancers, PDAC excludes antitumor T cells and maintains a dense population of immunosuppressive myeloid stroma (3). In addition, PDAC is highly hypoxic, a commonly cited characteristic associated with therapy resistance across multiple indications that has been shown to restrict effector T cell responses (4, 5).

Varied strategies have been pursued to therapeutically modulate oxygen tension within tumors, such as inhibiting oxidative phosphorylation to limit oxygen consumption, using antiangiogenics or hyperbaric oxygen chambers to improve oxygen delivery, and administering hypoxia-activated prodrugs to drive cellular remodeling of hypoxic regions (6–8). We have shown previously that the hypoxia-activated prodrug TH-302 (evofosfamide) can improve CD8^+^ T cell effector function and sensitize aggressive, spontaneous murine models of prostate cancer (9), as well as patients with immune-refractory cancer (10), to ICB therapy. Upon activation, TH-302 releases its cytotoxin, bromo-isophosphoramide mustard, which eliminates cells proliferating within tumor hypoxic regions and can trigger a vasculature remodeling cascade leading to both reoxygenation and restored T cell infiltration. Although this mechanism has been validated in melanoma, prostate cancer, lung cancer, and head and neck cancer (11, 12), some pancreatic cancers appear resistant to TH-302–mediated immune enhancement for reasons that are not understood (13). Antiangiogenic therapies have been shown to work in concert with TH-302 to combat tumor growth, as TH-302 is hypothesized to prevent exacerbated tumor hypoxia elicited by antiangiogenics (3, 14, 15). Notably, TH-302 with vascular endothelial growth factor receptor 2 (VEGFR-2) blockade induces endothelial cell apoptosis and improves radiotherapy responses in xenograft models of neuroblastoma. However, it is unclear if these findings extend to the poorly vascularized and hypoxic PDAC microenvironment and if the resulting environment becomes more hospitable to antitumor immunity with or without concomitant ICB.

Here, we studied how TH-302 and VEGFR-2 blockade act to remodel the PDAC microenvironment. We observed that the combination reduced hypoxic foci (but without improving overall tissue oxygenation), normalized the tumor vasculature, and led to increased intratumor DNA damage, which translated to an additive survival benefit over either monotherapy. Despite improved CD8^+^ T cell function, the addition of ICB failed to improve tumor responses to the combination. TH-302 and anti–VEGFR-2 (αVEGFR-2) did elicit greater granulocytic myeloid-derived suppressor cell (G-MDSC) infiltration into pancreatic tumors, however, as well as increased production of the tumor cell–derived chemokine CC chemokine ligand 9 (CCL9). These findings provide insight into how PDAC differs in response to hypoxia-activated, antiangiogenic immunotherapy compared with other cancers and point to G-MDSCs as key mediators of persistent immune resistance.

## Results

### Combination TH-302 and αVEGFR-2 treatment reduces hypoxic foci and normalizes the PDAC tumor vasculature.

**Figure 1:**
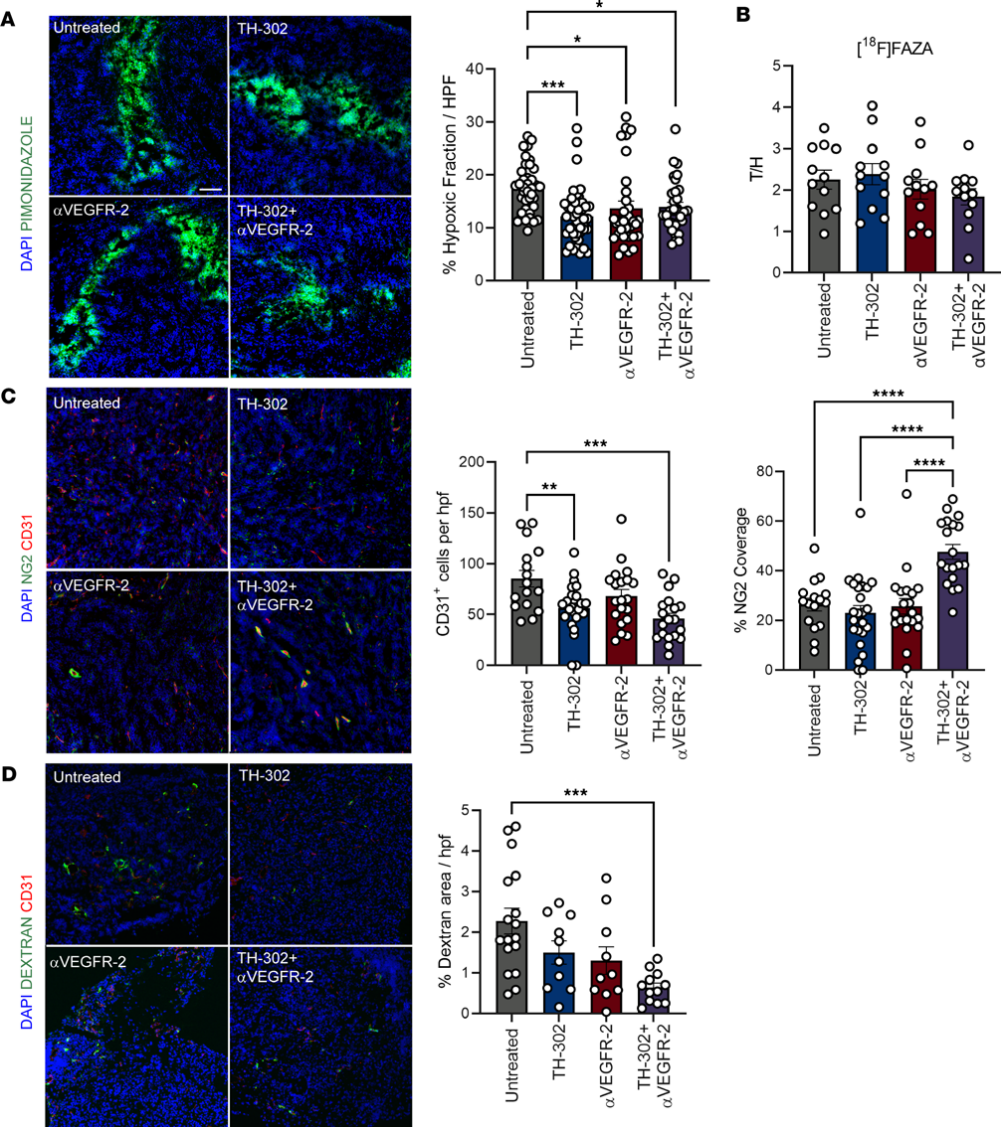
Hypoxia-activated TH-302 with antiangiogenic αVEGFR-2 therapy reduces hypoxic foci and reorganizes the tumor vasculature. Mice bearing 10-day established orthotopic pancreatic tumors were treated with TH-302 for 5 consecutive days and received 2 doses of αVEGFR-2 administered once every 4 days. On day 15 after tumor challenge, mice were injected with pimonidazole or dextran, and tumors were harvested for optimal cutting temperature (OCT) embedding. (**A**) Percentage area per high-power field (hpf) of pimonidazole staining in MT4-LA tumors and representative images (*n* = 4–6 per group, 4–12 hpf per tumor). (**B**) [^18^F]FAZA retention in tumors versus heart (T/H) (*n* = 12 per group). (**C**) Absolute count of CD31^+^ cells, percentage CD31 colocalized with neuron-glial antigen 2 (NG2), and representative images (*n* = 4–6 per group, 3–5 hpf per tumor). (**D**) Percentage area of FITC-dextran and representative images. Scale bar represents 100 μm (*n* = 3–6 per group, 1–5 hpf per tumor). (**A**, **C**, and **D**) Repeated measures 1-way ANOVA followed by Tukey’s correction for multiple comparisons, *P adj < 0.05, **P adj < 0.01, ***P adj < 0.001, ****P adj < 0.0001; data are mean ± SEM.

To test the hypothesis that TH-302 would complement antiangiogenic therapy, we used the hypoxia-activated prodrug TH-302 and an antibody targeting VEGFR-2, which can transiently improve tumor oxygenation and normalize tumor vasculature (16). Given that both modalities work through distinct mechanisms, we used immunofluorescence to evaluate each therapy’s independent effects on hypoxia and tumor vasculature in orthotopically implanted MT4-LA, a luciferase expressing mT4-2D derived pancreatic cancer cell line that was isolated from *Kras^+/G12D^ TP53^+/R172H^ Pdx1-Cre* organoid cultures that models the poor vascularity of human pancreatic cancer and is unresponsive to standard-of-care chemotherapy (17–19). We observed decreased regions of hypoxic foci, labeled by pimonidazole, after monotherapy and combination treatment; however, total oxygenation status measured by ^18^F-fluoroazomycin arabinoside ([^18^F]FAZA) remained insignificantly changed (Figure 1, A and B). Tumor endothelial cells, marked by CD31 staining, were reduced by TH-302 treatment (Figure 1C and Supplemental Figure 1; supplemental material available online with this article; https://doi.org/10.1172/jci.insight.169150DS1). Additionally, tumor endothelial cells showed an increase in cleaved caspase-3 without any significant shifts in Ki67 expression with TH-302 and αVEGFR-2 (Supplemental Figure 1). We next asked if the remaining tumor vessels were stable and functional by probing for NG2 colocalization with CD31-positive endothelial cells and measuring vessel leakage. We observed increased colocalization of NG2 and CD31 as an indication of improved vessel pericyte coverage in the combination treatment group compared with either monotherapy (Figure 1C). Furthermore, we observed decreased dextran in combination-treated tumors, indicating reduced vessel leakage (Figure 1D). Put together, we concluded that hypoxia reduction was regionally restricted within the tumor, while the tumor vasculature experienced pruning of immature vessels, thereby leaving mature, more functional endothelium.

### TH-302 and αVEGFR-2 therapies provide an additive survival benefit in orthotopic pancreatic and transplantable prostate models.

**Figure 2:**
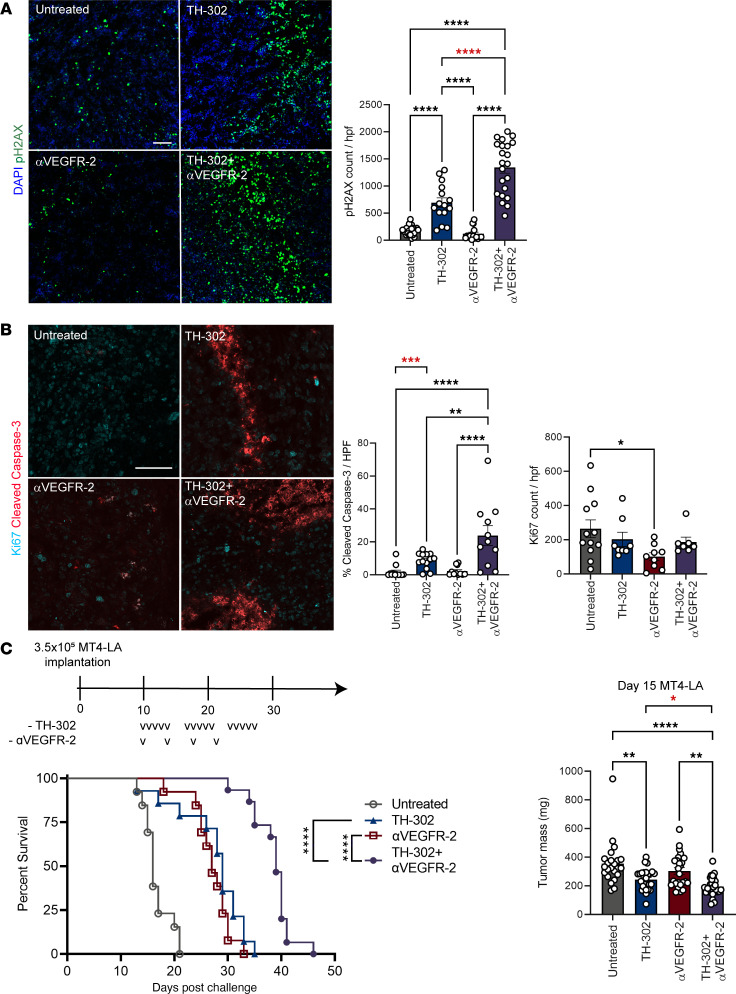
TH-302 cooperates with αVEGFR-2 to extend survival of mice with pancreatic and prostate tumors. (**A** and **B**) Ten-day established orthotopic pancreatic tumors were resected from mice 24 hours after completion of 5 consecutive treatments of TH-302 and 2 doses of VEGFR-2 antibody spaced 4 days apart for OCT embedding and sectioning. (**A**) Phosphorylated H2AX (pH2AX) foci counts and representative images (*n* = 3–5 per group, 2–7 hpf per tumor) and (**B**) percentage area of cleaved caspase-3 and Ki67 foci counts per hpf (*n* = 3–5 per group, 1–3 hpf per tumor). (**C**) (Left) Survival (*n* = 12–15 per group) and (Right) tumor burden (*n* = 23–27 per group) of mice bearing orthotopic MT4-LA pancreatic tumors. Tumor burden was measured by weight after a 5-day cycle of TH-302 and 2 doses of VEGFR-2 antibody blockade therapy. Lowercase Vs represent doses. Scale bar represents 100 μm. (**A**, **B**, and **C** right) Repeated measures 1-way ANOVA followed by Tukey’s correction for multiple comparisons, (**C** left) log-rank (Mantel-Cox) test. Red asterisks indicate analysis by unpaired 2-tailed Student’s *t* test applied to the 2 indicated groups. *P adj < 0.05, **P adj < 0.01, ***P adj < 0.001, ****P adj < 0.0001; data are mean ± SEM.

Given these observations, and that vessel leakage is associated with poor delivery of therapeutics, we sought to determine if the combination strategy led to a greater number of phosphorylated histone H2AX (p-H2AX) foci formation, an indication of TH-302–induced DNA damage and, consequently, greater antitumor activity (20). Formation of p-H2AX foci was markedly increased after TH-302 treatment, with the combination therapy eliciting the greatest amount of DNA damage and cleaved caspase-3, while Ki67 remained comparable except for the reduced foci seen with αVEGFR-2 monotherapy (Figure 2, A and B).

To determine if the increased DNA damage and cell death translated to greater antitumor activity, orthotopic MT4-LA pancreatic tumors were established prior to mice receiving three 5-day cycles of TH-302 treatment, 4 doses of αVEGFR-2, or the combination. We found monotherapy with TH-302 or αVEGFR-2 increased median overall survival (mOS) to 29 and 27 days, respectively, while the combination led to mOS of 39 days and reduced tumor burden (Figure 2C). This benefit could also be reproduced using low-dose αVEGFR-2 treatment (Supplemental Figure 2A). The additive benefit extended to prostate TRAMP-C2A (an aggressive subclone of TRAMP-C2) tumors, wherein the combination group experienced an increased mOS (50 days) over either monotherapy and a significant delay in tumor growth (Supplemental Figure 2, B and C). We next sought to determine if the survival benefit conferred by TH-302 and VEGFR-2 was reliant on an enhanced adaptive immune response. However, we observed wild-type mice and recombination-activating gene–knockout mice treated with the combination scheme had comparable survival rates (Supplemental Figure 3). These data suggest that the combination of hypoxia-activated chemotherapy and blockade of VEGFR-2 signaling effectively combats both pancreatic and prostate tumors but does so without engaging or promoting enhanced adaptive antitumor immunity.

### Programmed cell death protein 1 blockade fails to improve pancreatic tumor response to TH-302 and αVEGFR-2.

**Figure 3:**
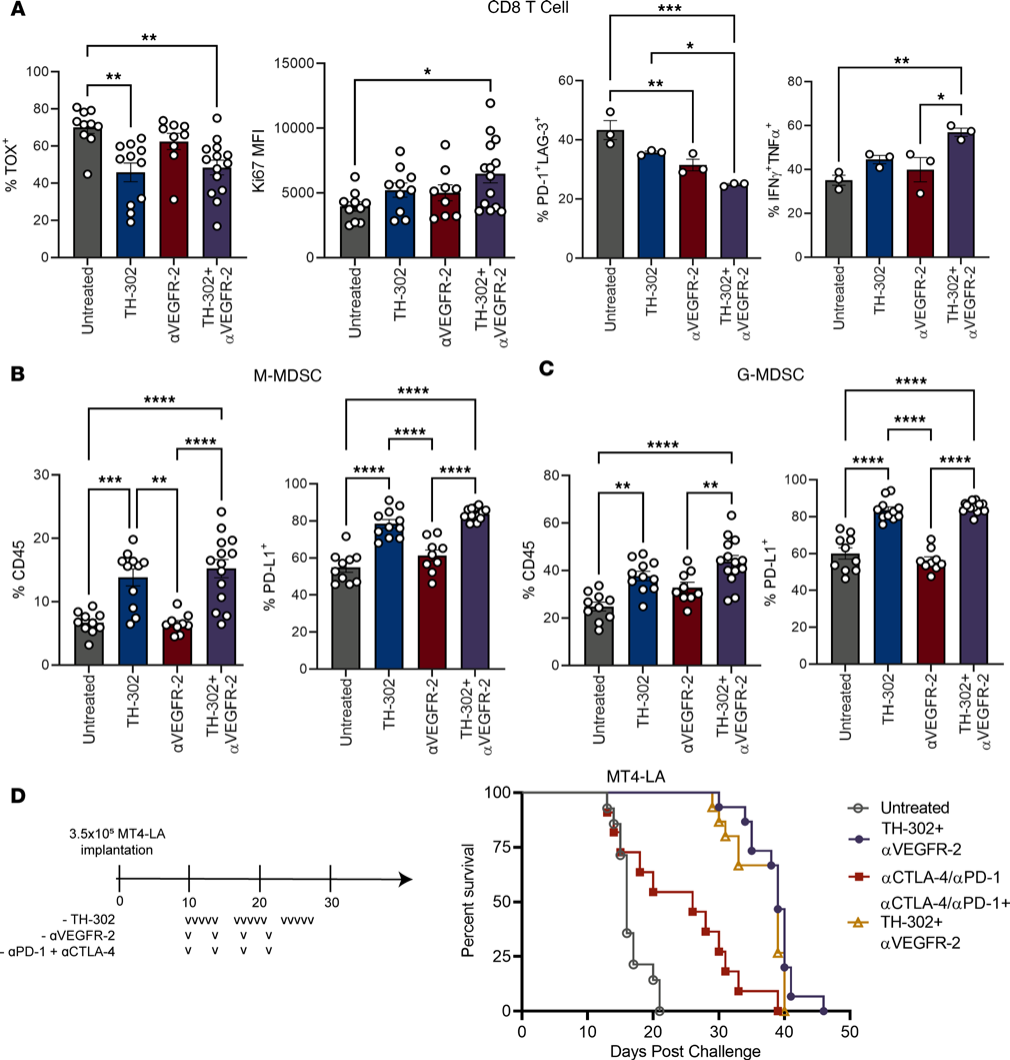
ICB therapy fails to improve therapeutic responses to combination TH-302 and αVEGFR-2. Mice bearing 10-day established MT4-LA tumors were treated for 5 consecutive days with TH-302 and 2 doses of VEGFR-2 spaced 4 days apart. On day 15, tumors were resected and processed for analysis of tumor-infiltrating immune cells. (**A**) CD8^+^ T cells were analyzed for expression of Ki67 and TOX (*n* = 10–14 per group). Poststimulation, CD8^+^ T cells were analyzed for expression of interferon-γ (IFN-γ), tumor necrosis factor-α (TNF-α), PD-1, and LAG-3 (*n* = 4–5 tumors pooled per group, 3 independent experiments). Frequency and percentage of PD-L1–expressing (**B**) M-MDSCs and (**C**) G-MDSCs (*n* = 10–14 per group). (**D**) Survival of mice bearing orthotopic MT4-LA treated with TH-302, αVEGFR-2, αPD-1, and αCTLA-4. Untreated mice and those treated with combination TH-302 and αVEGFR-2 are first presented in Figure 2 (*n* = 10–14 per group). Lowercase Vs represent doses. (**A**–**C**) One-way ANOVA followed by Tukey’s correction for multiple comparisons. (**D**) Log-rank (Mantel-Cox) test. *P adj < 0.05, **P adj < 0.01, ***P adj < 0.001, ****P adj < 0.0001; data are mean ± SEM.

Both hypoxia and dysfunctional vasculature negatively affect CD8^+^ T cell effector function and are associated with poor responses to ICB (4, 21, 22). Using flow cytometry, we examined how TH-302 and αVEGFR-2 treatment affects the tumor immune infiltrate. While the percentage of total immune infiltrate did not change with combination treatment versus that of untreated counterparts (Supplemental Figure 4A), we found that the frequency of CD8^+^ T cells coexpressing markers of dysfunction, such as programmed cell death protein 1 (PD-1) and lymphocyte-activation gene 3 (LAG-3), and the exhaustion master transcription factor, thymocyte selection-associated high mobility group box (TOX), were reduced with TH-302 treatment (Figure 3A). Additionally, CD8^+^ T cells expressed more Ki67, and a greater proportion gained multipotent cytokine competency when treated with the combination of TH-302 and VEGFR-2 blockade (Figure 3A). These phenotypic changes in CD8^+^ T cells are consistent with previous reports demonstrating that reversal of hypoxia and normalization of tumor vasculature improve antitumor CD8^+^ T cell function (7, 9, 23–26).

Unexpectedly, we observed that delivery of TH-302 expanded the G-MDSC and monocytic myeloid-derived suppressor cells (M-MDSC) compartments without having a significant impact on the frequency of total tumor-infiltrating immune cells (Supplemental Figure 4, A and B, and Figure 3, B and C). Of the G-MDSCs that infiltrated the tumor, nearly all expressed Arginase-1, an indicator of their suppressive state that helps differentiate them from N1 tumor-associated neutrophils (TANs) that can have antitumor functionality (Supplemental Figure 4C) (27). In contrast, CD8^+^ T cells, dendritic cells (DCs), regulatory T cells (Tregs), and tumor-associated macrophages (TAMs) experienced a reduction in frequency upon combination TH-302 and αVEGFR-2 therapy compared with their untreated counterparts (Supplemental Figure 4D). Additionally, a greater frequency of MDSCs expressed programmed cell death protein ligand 1 (PD-L1) after TH-302 treatment (Figure 3, B and C, and Supplemental Figure 4E), which was a shared characteristic among Tregs and DCs (Supplemental Figure 4E). In a similar fashion, TAMs increased their PD-L1 expression in response to combination therapy (Supplemental Figure 4E).

Given the increased frequency of cells expressing PD-L1 and the improved CD8^+^ T cell response conferred by combination TH-302 and αVEGFR-2 therapy, we hypothesized that the addition of ICB could further enhance the therapeutic response by expanding and functionally protecting infiltrating T cells. However, using both PD-1–blocking and cytotoxic T lymphocyte–associated protein 4–blocking (CTLA-4–blocking) antibodies in combination with TH-302 and αVEGFR-2 failed to improve tumor responses (Figure 3D). In contrast, αCD40 provided an additive benefit when combined with the combination or either monotherapy (Supplemental Figure 6). Taken together, these data indicated that myeloid-derived mechanisms were involved in restricting ICB responses in the context of vascular normalization and that a CD40 agonist antibody could partially alleviate CD8^+^ T cell suppression.

### Neutrophil-derived genes are enriched in combination TH-302 and αVEGFR-2 treated tumors.

**Figure 4:**
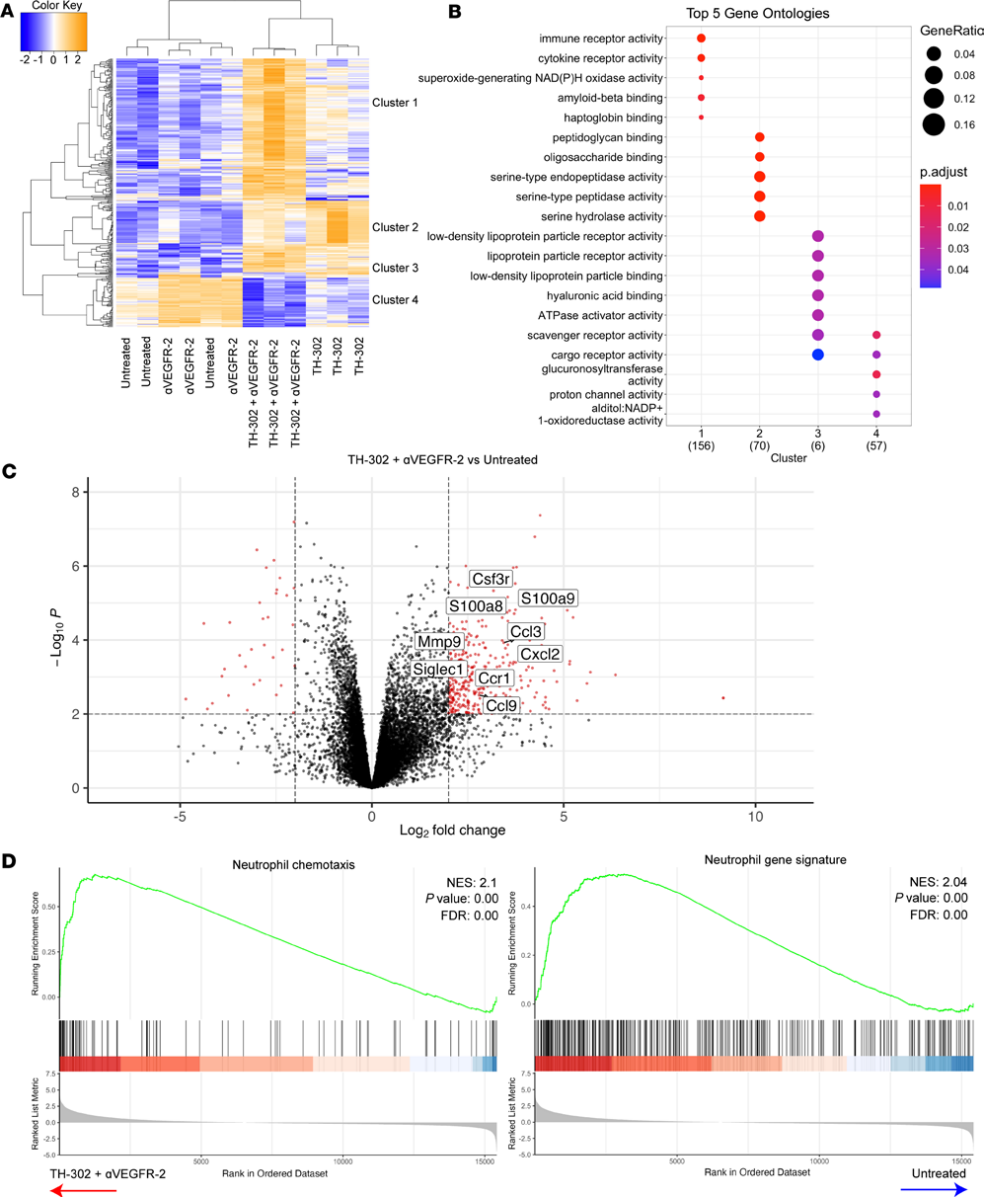
Combination TH-302 and αVEGFR-2 treated tumors are enriched with a neutrophil gene signature. Tumors were resected on day 15 after a 5-day cycle of TH-302 and 2 αVEGFR-2 treatments before whole tumors were processed for RNA isolation followed by RNA sequencing and transcriptome analysis. (**A**) Heatmap and unsupervised clustering of 327 differentially expressed genes (|fold-change| > 1.5 and FDR < 0.01) and (**B**) gene ontology analysis of genes defining each cluster represented in a bubble plot. Numbers in parentheses represent the number of genes in each cluster. (**C**) Volcano plot representing log fold-change gene expression of combination TH-302 and αVEGFR-2 treated tumors versus untreated. Red dots indicate differentially expressed genes that reach statistical significance threshold and fold-change thresholds. Black dots represent genes that do not. (**D**) Leading edge plots of neutrophil chemotaxis and neutrophil gene signature. (**A**–**D**) *n* = 3 per group. NES, normalized enrichment score.

We next sought to determine the mechanism(s) by which MT4-LA tumors resisted response to ICB in the context of TH-302 and αVEGFR-2 therapy. To do this, we analyzed RNA-sequencing (RNA-Seq) data from untreated or combination TH-302 and αVEGFR-2 treated tumors. Unsupervised hierarchical clustering of differentially expressed genes showed that the combination treatment drove a transcriptional program distinct from that of each monotherapy (Figure 4A). Genes driven by the combination of TH-302 and αVEGFR-2 (cluster 1) were associated with immune and cytokine receptor activity and NADPH oxidase activity, of which the latter is associated with reactive oxygen species generation in neutrophils (Figure 4B) (28). Genes that clustered and were shared with TH-302 programming were associated with serine-type endopeptidase activity and polysaccharide binding (Figure 4B).

Analysis of differentially expressed genes in combination TH-302 and αVEGFR-2 treatment showed enrichment of genes associated with neutrophils and G-MDSCs (Figure 4C and Supplemental Figure 7A). Gene set enrichment analysis (GSEA) suggested that combination TH-302 and αVEGFR-2 resulted in elevated neutrophil infiltration, increased epithelial cell apoptosis, and decreased oxidative phosphorylation (Figure 4D and Supplemental Figure 7B). Combined, these data suggested that combination treatment led to tumor cell death and modulated the influx of pathologically activated neutrophils.

### TH-302 and αVEGFR-2 combination treatment enhances myeloid cell mobilization toward pancreatic tumors.

**Figure 5:**
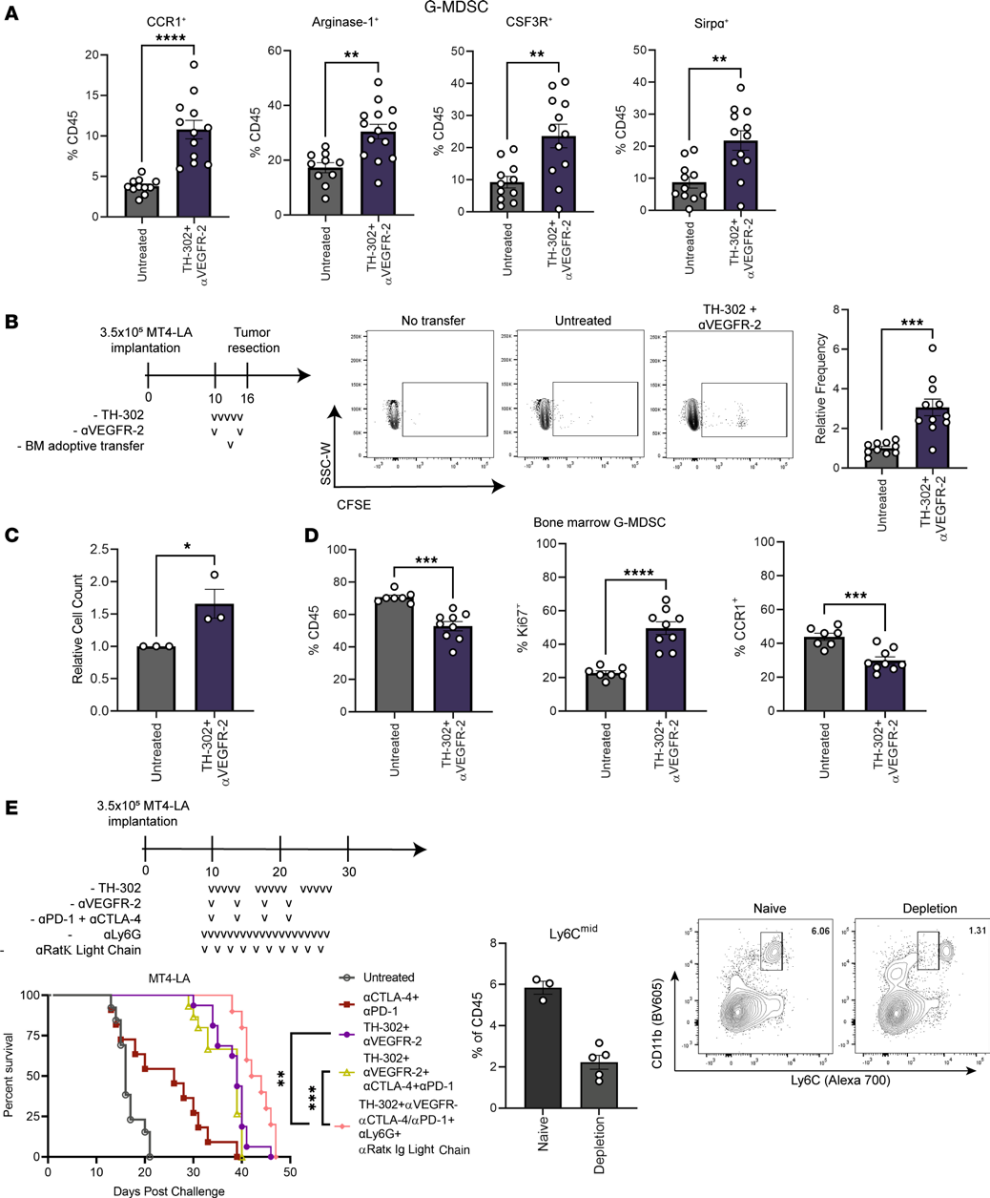
G-MDSCs readily migrate into TH-302 and αVEGFR-2 treated tumors. Mice challenged with MT4-LA were treated with TH-302 and VEGFR-2 blockade antibody as described earlier before tumors were resected and processed for flow cytometry analysis of tumor-infiltrating immune cells. (**A**) Frequency of CD45^+^ cells composed of G-MDSCs expressing CCR1, Arginase-1, CSF3R, and SIRPα (*n* = 11–12 per group). (**B**) Relative frequency of CD11b^+^Ly6G^+^CFSE^+^ cells present in the tumor following adoptive transfer (*n* = 10–11 per group). Lowercase Vs represent doses. SSC-W, side scatter width. (**C**) Relative numbers of Ly6G^+^ cells that migrated toward lysate derived from untreated or combination TH-302 and αVEGFR-2 treated tumors (*n* = 4–5 per group, 3 independent experiments). (**D**) Bone marrow cells from pancreatic tumor–bearing mice were analyzed for expression of Ki67, CXCR4, CXCR2, and CCR1 (*n* = 7–9 per group). (**E**) Left: Survival of mice treated with a dual-antibody strategy for depleting Ly6G^+^ cells. Lowercase Vs represent doses. Right: Peripheral blood from naive (staining control) and treated mice was assessed for depletion of G-MDSCs (CD11b^+^Ly6C^mid^) on day 16. Untreated, TH-302 and αVEGFR-2, and their combination with CTLA-4/PD-1 antibody blockade are first presented in Figure 3. Numbers in upper-right corners represent the frequency of the parent gate (CD45^+^ cells). (**A**–**D**) Unpaired 2-tailed Student’s *t* test. (**E**) Log-rank (Mantel-Cox) test. *P adj < 0.05, ***P adj < 0.001, ****P adj < 0.0001; data are mean ± SEM.

To validate the enriched neutrophil gene signature, we analyzed CD11b^+^Ly6G^+^Ly6C-midlevel (^mid^) cells from the bone marrow and tumors of mice and determined the propensity of naive bone marrow–derived cells to migrate toward treated tumors. Twenty-four hours after a single cycle of combination TH-302 and αVEGFR-2 therapy, we observed a greater percentage of CD45-positive immune infiltrate composed of G-MDSCs expressing CC chemokine receptor 1 (CCR1), Arginase-1, colony stimulating factor 3 receptor (CSF3R), and signal regulatory protein alpha (SIRPα) (Figure 5A). We additionally observed increased levels of matrix metalloproteinase 9 (MMP9), a potential indicator of G-MDSC presence (Supplemental Figure 8). Since the GSEA indicated increased G-MDSC infiltration upon treatment, we adoptively transferred naive bone marrow cells labeled with carboxyfluorescein diacetate succinimidyl ester (CFSE) and determined their frequency in the tumor after 60 hours. Pancreatic tumors in mice treated with TH-302 and αVEGFR-2 demonstrated a nearly 3-fold greater capacity to accumulate adoptively transferred Ly6G^+^ cells than their untreated counterparts (Figure 5B). This finding was corroborated by in vitro chemotaxis studies of bone marrow cells that demonstrated enhanced migration toward bulk tumor–derived lysate from treated mice and by immunofluorescence images of treated tumors showing the presence of more Ly6G^+^ cells localized outside of areas that accumulated DNA damage (Figure 5C and Supplemental Figure 9). Additionally, the frequency of Ly6G^+^ cells in the bone marrow of treated mice was reduced despite the increased percentage of cells expressing Ki67, potentially signifying egress of neutrophils in response to tumor chemokine release (Figure 5D). When chemokine receptor expression was assessed, we found that the frequency of CCR1-expressing bone marrow Ly6G^+^ cells contracted versus those expressing CXC chemokine receptor 2 (CXCR2) or CXCR4 (Figure 5D). Put together, these data suggest that Ly6G^+^ cells in the bone marrow of TH-302 and αVEGFR-2 treated mice actively proliferate and exit the bone marrow via CCR1 toward the tumor in response to combination TH-302 and VEGFR-2 blockade treatment. To determine if G-MDSCs mediate resistance to ICB, we performed in vivo depletion using a double-antibody strategy against Ly6G and Rat-kappa light chain (29). Mice that received G-MDSC depletion with TH-302, VEGFR-2, and ICB achieved a significant improvement in survival over their nondepleted counterparts (Figure 5E).

### Tumor-derived CCL9 is elevated in dual-modality–treated tumors.

**Figure 6:**
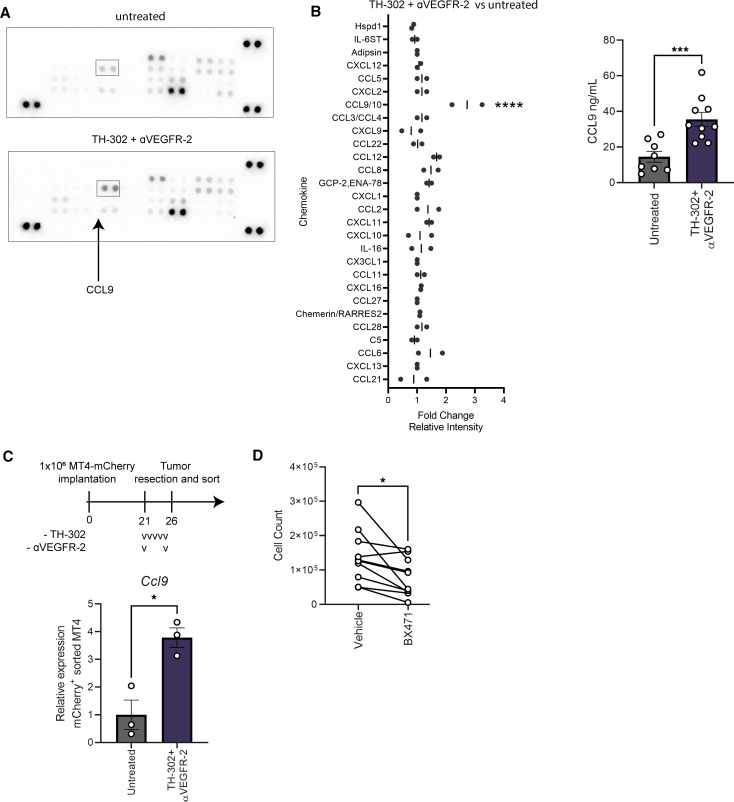
CCL9 expression is induced upon combination TH-302 and αVEGFR-2 therapy. (**A** and **B**) Bulk tumor lysate was extracted from 10-day established MT4-LA tumors resected on day 15 from mice that received 5 consecutive days of TH-302 treatment and 2 αVEGFR-2 treatments spaced 4 days apart. (**A**) Representative mouse chemokine array membranes incubated with bulk tumor lysate (*n* = 2–3 pooled tumors per group, 2 independent experiments). (**B**) Tumor lysates were tested for the presence of CCL9 by ELISA (*n* = 8–10 per group). (**C**) mCherry^+^ tumor cells sorted from orthotopic pancreatic tumors established for 21 days, treated with 1 cycle of TH-302 and 2 doses of αVEGFR-2 antibody, and then assessed for relative *Ccl9* transcript by quantitative PCR (qPCR) (normalized to *Hprt*, *n* = 2–3 pooled tumors per sample per group). Lowercase Vs represent doses. (**D**) Cell count of vehicle- or 20 μM BX471–treated Ly6G^+^ cells that migrated toward combination TH-302 and αVEGFR-2 treated tumor-derived lysate (*n* = 10 per group). (**B**–**D**) One-way ANOVA followed by Tukey’s correction for multiple comparisons. *P adj < 0.05, **P adj < 0.01, ***P adj < 0.001, ****P adj < 0.0001; data are mean ± SEM.

We next asked which secreted factor may be responsible for the increased accumulation of G-MDSCs in combination-treated tumors. In alignment with the RNA-Seq analysis, CCL9 was enriched in lysates from TH-302 and αVEGFR-2 treated tumors (Figure 6, A and B). To determine the tumor cell–intrinsic production of CCL9, we analyzed RNA from FACS-sorted, mCherry-labeled MT4 tumors and found *Ccl9* was upregulated in treated tumor cells (Figure 6C and Supplemental Figure 10). To determine if CCL9 was the major chemokine responsible for regulating G-MDSC recruitment, we used an antagonist compound, BX471, to inhibit the receptor for CCL9, CCR1 (30–32). Compared with vehicle-treated cells, those treated with BX471 had a limited ability to migrate toward lysates collected from combination TH-302 and VEGFR-2 blockade treated tumors (Figure 6D). Last, interrogation of The Cancer Genome Atlas (TCGA) pancreatic adenocarcinoma (PAAD) cohort showed that both *CCR1*, the cognate receptor for *Ccl9*, and *CCL23*, the human homolog of murine *Ccl9* (33), correlated with a TAN gene signature (Supplemental Figure 11). Together, these data indicate that the CCL9/CCR1 axis probably plays a role in the influx of G-MDSCs into the tumor after treatment with TH-302 and VEGFR-2 blockade.

## Discussion

Here, we report the additive benefit of combining the hypoxia-activated prodrug TH-302 with the antiangiogenic αVEGFR-2 in extending the survival of mice with pancreatic cancer. Our study revealed that the combination therapy elicited normalization of the tumor vasculature and accumulation of DNA damage that resulted in tumor debulking and was associated with a qualitatively better CD8^+^ T cell response. Despite these changes, the addition of ICB failed to enhance the therapeutic benefit of the drugs alone. Our analysis of these data revealed multiple potential resistance mechanisms that warrant further investigation, including the lack of complete hypoxia reduction despite the increased density of DNA damage elicited by TH-302 and the increased accumulation of G-MDSCs that block ICB expansion of T cell immunity following treatment.

Earlier studies demonstrated the promise of hypoxia reduction using TH-302 as a single agent across multiple human xenograft and syngeneic murine models of cancer (34). The activity of TH-302 is reported to extend beyond hypoxic regions to normoxic areas, termed the “bystander effect”; however, definitive data regarding if active TH-302 metabolites can diffuse or if TH-302 is activated under oxygenated conditions remain elusive (35–37). Further, tissue reoxygenation after TH-302 therapy may be driven by reduced oxygen consumption, as suggested by decreased perfusion posttreatment; by TH-302 inhibiting mitochondrial respiration at complexes I, II, and IV; and by the combination regimen of TH-302 and VEGFR-2 blockade leading to a unenriched oxidative phosphorylation signature (13, 38). Despite these observations, TH-302 alone and in combination with αVEGFR-2 failed to increase total oxygen availability in the tumor, though hypoxic foci were reduced. We interpret these data to suggest that MT4-LA is partially resistant to TH-302 and exhibits a similar response as other tumor lines, such as pancreatic Su86.86 and renal cell carcinoma 786-O, where oxygen levels do not change with treatment (13, 39, 40). Interestingly, we observed a similar insensitivity to TH-302 with the prostate cancer model TRAMP-C2A with regard to tumor growth, which could be partially overcome when TH-302 was combined with VEGFR-2 blockade and thus shows the potential application of this combination therapy scheme across models. Whether the lack in curative responses observed with both models is due to vessel pruning by TH-302 or the active conversion of TH-302 within normoxic regions as suggested by the accumulation of p-H2AX foci across the length of imaged fields (Figure 2A) or represents a block to a necessary second step within the tissue that promotes normal neovascularization remains to be explored further.

We found that TH-302 with αVEGFR-2 enhanced the functional properties of the CD8^+^ T cell response, in line with previous data demonstrating that each monotherapy alone can improve immunotherapy responses (9, 11, 24, 41, 42). The enhanced multipotent cytokine competency and reduced frequency of CD8^+^ T cells expressing markers of dysfunction observed here may result from the antiangiogenic effects and improved metabolic environment driven by the dual combination, as increased antitumor control and improved T cell responses have been observed across preclinical models using various antiangiogenic therapies (23–25, 43). Further, the PD-L1 upregulation observed in this study is seen with the use of VEGFR-2 blockade and dual angiopoietin-2 and VEGF-A inhibition (23, 24). Further work is necessary to confirm whether PD-L1 upregulation occurs in response to increased IFN-γ production or, potentially, as a result of DNA double-strand break repair pathway activation (44).

Despite the functional enhancement CD8^+^ T cells gained, ICB therapy failed to improve tumor responses. This probably resulted in part from the paucity of CD8^+^ T cells observed after treatment with the combination of TH-302 and VEGFR-2 blockade, which may be driven by the concomitant increase in the proportion of G-MDSCs after treatment with TH-302. In contrast with ICB, CD40 agonist therapy provided an additive benefit when combined with TH-302, αVEGFR-2, or the combination, which suggests softening of MDSC-mediated immune suppression. Other mechanisms by which CD40 agonists protect or enhance the CD8^+^ T cell response may also be engaged, such as enhanced antigen presentation and reduced collagen deposition (45, 46); however, further work is necessary to understand how CD40 is working in concert with TH-302 and VEGFR-2 blockade therapy to improve mouse survival.

RNA-Seq of bulk tumor samples verified an enrichment of neutrophil-derived genes in combination-treated tumors, specifically, genes associated with pathological activation of neutrophils and MDSC-mediated suppressive function, such as *S100a8/a9*, *Mmp9*, and *Arg1*. Initially, we were surprised to find that the percentage of G-MDSCs expressing Arginase-1, CCR1, CSF3R, and SIRPα remained unchanged (AL, unpublished observations); however, the frequency of G-MDSCs expressing these markers as a proportion of the total immune compartment increased, suggesting that the combination therapy scheme modulates the cellular composition of tumors, rather than increasing the suppressive function of each cell. Flow cytometry data corroborated these findings and suggested that TH-302 alone increased the frequency of G- and M-MDSCs present in pancreatic tumors. How this occurs, whether through increased recruitment, expansion, or enhanced survival ability, and how TH-302 influences these processes, remain to be explored. In the combination treatment setting, the increased frequency of G-MDSCs likely arises from the enhanced recruitment of bone marrow–derived cells that occurs in part through the CCR1/CCL9 signaling axis. This hypothesis is supported by our findings that a reduced proportion of CCR1-expressing myeloid cells remain in the bone marrow, by the treated versus untreated differential gene expression, by ELISA of bulk tumor lysates, and by the observation that qPCR of tumor cells pointed to CCL9 as differentially produced in combination-treated tumors. Furthermore, blockade of the CCR1/CCL9 axis limited G-MDSC trafficking toward lysates derived from combination-treated tumors (Figure 6D). Our findings implicate G-MDSCs as part of an adaptive response to vascular-normalizing therapy given that their increased infiltration is associated with reduced endothelial cell density, vessel leakiness, and changes to endothelial pericyte coverage. These observations complement previous work depicting MDSCs as mediators of resistance to antiangiogenic therapies through their ability to secrete vascular-remodeling factors, such as MMP9 and S100A8/A9, and corroborate the production of CCL9 as a mediator of immune resistance in pancreatic cancer (47–51).

Recruitment of MDSCs to the tumor microenvironment via tumor cell–intrinsic factors and their ability to suppress antitumor immunity are well documented. Induction of CCL9-induced myeloid cell recruitment has been previously reported and reflects one of multiple secreted factors that regulate the accumulation of myeloid cells (52, 53). In pancreatic tumors, CXCL1 in concert with CSF3 facilitate G-MDSC infiltration and establishment of an immunosuppressive microenvironment that limits T cell frequency and function (54). Furthermore, CXCL2, CXCL12, CCL2, and CXCL8 in humans have each been observed to regulate G-MDSC recruitment to the tumor microenvironment (27). While the chemokine array data indicated CCL9 as a strong candidate, we cannot discount other changes in chemokine and chemokine receptor transcript levels in response to therapy, including *Cxcr4*, *Cxcl2*, and *Ccl3* (Figure 4C and Supplemental Figure 7). These observations suggest that multiple factors work together to recruit and maintain a dense G-MDSC population following therapy. Further investigation of the upstream regulators that drive chemokine production in tumor cells is warranted and is likely to provide new therapeutic opportunities once uncovered.

Importantly, our observations in pancreatic cancer presented here differ from our work in prostate cancer, whereby TH-302 catalyzed the reduction of suppressive MDSCs and compromised the ability of tumors to recruit MDSCs through its hypoxia-eliminating capabilities (9). This hypoxia-reducing effect did not translate to the pancreatic cancer setting, even when combined with αVEGFR-2 antibody, and failed to generate a potent adaptive immune response capable of inducing tumor rejection. These observations suggest that the persistent hypoxic state remaining posttreatment may nurture a tumor microenvironment that favors immunosuppressive myeloid cell recruitment and continues to limit CD8^+^ T cell expansion. Future work will determine if optimization of therapeutic strategies that alleviate hypoxia more completely will diminish MDSC density in pancreatic cancer.

In conclusion, this study enhanced our current understanding of how hypoxia-activated chemotherapy works in concert with antiangiogenic therapy. Our results indicate that the combination therapy leads to vascular normalization and increased DNA damage that translates to greater tumor control. While CD8^+^ T cells gain enhanced functional capabilities, ICB fails to provide an additive benefit. In response to the combination therapy, we find that tumor cells increase production of CCL9, which is associated with greater mobilization of G-MDSCs out of the bone marrow and into the tumor bed, which could be limited by CCR1 inhibition. The extension of these observations to the clinic remains to be studied; currently, TH-302 is being evaluated in combination with bevacizumab in bevacizumab-refractory glioblastoma, where the combination is reported to provide increased median time to progression relative to historical data (14). Our findings suggest that G-MDSC recruitment is an adaptive response that mediates resistance to otherwise hypoxia-normalizing therapies at the level of both normoxia restoration and infiltrating T cell expansion.

While we observed a survival benefit conferred by TH-302 and VEGFR-2 in an orthotopic, transplantable murine model of pancreatic cancer and identified the CCL9/CCR1 axis as responsible for the recruitment of G-MDSCs to the tumor microenvironment posttherapy, we acknowledge that these conclusions are drawn from 1 pancreatic tumor cell line. Additionally, our observation of increased G-MDSC influx under persistent hypoxia remains to be validated in patient cohorts treated with TH-302, VEGF-A/VEGFR-2 inhibition, or other hypoxia-modifying therapeutic strategies.

## Methods

### Mice.

Male C57BL/6J and B6.129S7-*Rag1^tm1Mom^*/J mice were purchased from The Jackson Laboratory. Mice were housed in our Association for Assessment and Accreditation of Laboratory Animal Care International–accredited, pathogen-free facility. Mice were used between 5 and 10 weeks of age.

### Cell lines.

MT4-LA was generated and maintained as reported (33). MT4-Cherry cells were generated by retroviral transduction of a pMG-mCherry-luciferase vector that is based on pGC-IRES (55) into the Tuveson lab–derived mT4 PDAC cell line (Cold Spring Harbor Laboratory). TRAMP-C2A is an aggressive subclone derived from the TRAMP-C2 (provided by Norman Greenberg, Baylor College of Medicine, Houston, Texas, USA) tumor cell line and was cultured as previously described (9).

### Syngeneic tumor models.

We injected 3.5 × 10^4^ MT4-LA cells in 50 μL of 30% Matrigel (Corning, 356231) into the head of the pancreas using a U-40 insulin syringe fitted with a 29.5-gauge needle (17). A total of 1 × 10^6^ TRAMP-C2A cells were injected on the subcutaneous flank in 100 μL of phosphate-buffered saline (PBS). Tumor volume and death were recorded as described previously (56).

### Antibody and TH-302 treatment.

Therapeutic antibodies for in vivo treatment were obtained from Leinco Technologies or Bio X Cell. A total of 4 mg/kg of αCTLA-4 (clone 9H10), 4 mg/kg of αCD40 (clone FGK45), 10 mg/kg of αPD-1 (clone RMP1-14), and 10 or 40 mg/kg of αVEGFR-2 (clone DC101) were delivered via intraperitoneal (IP) injection. TH-302 was provided by Molecular Templates, Inc. and administered IP at 50 mg/kg.

### Immunofluorescence.

Mouse tissues were resected as described under *Flow cytometry* after completion of 1 cycle of therapy. Processing of tissue was described previously, with the exception that fixation was done using the Foxp3/Transcription Factor Fixation/Permeabilization kit (Invitrogen, 00–5521-00) for 20 minutes and blocked with 2% bovine serum albumin (BSA) and 25 μg/mL 2.4G2 antibody (Leinco Technologies Inc., C381) in Tris-buffered saline with Tween 20 (TBS-T) for 1 hour at 20°C (9). Samples were stained with antibodies in 2% BSA in TBS-T overnight at 4°C. Pimonidazole (Hypoxyprobe, HP6-200Kit) was administered via IP injection 1.5 hours before mouse euthanasia. A total of 10 mg/mL of 2,000,000 average MW dextran (MilliporeSigma, FD2000S) was i.v. injected 10 minutes before mice were sacrificed. Tissue sections were imaged using a Carl Zeiss LSM 780 NLO with a plan-apochromat of 10×/0.45 or 20×/0.8.

### Flow cytometry.

Samples were processed and analyzed as previously described (57). Pancreatic tumors were processed 24 hours after completion of the first cycle of therapy: 5 doses of TH-302 administered once daily and 2 doses of αVEGFR administered every 4 days. The pellet from the Histopaque-1119 (MilliporeSigma, 11191) separation was processed for endothelial cell analysis. CD8^+^ T cells were isolated using a magnetic-activated cell sorting CD8a^+^ T Cell Isolation Kit (Miltenyi Biotec, 130-104-075), activated for 6 hours using the eBioscience Cell Stimulation Cocktail plus protein transport inhibitors (Thermo Fisher Scientific, 00-4975-93), and analyzed by flow cytometry as previously described (56). Flow data were collected on a 5-laser, 18-color BD LSR II and analyzed using FlowJo version v10.7. A representative illustration of the immune cell gating scheme is provided in Supplemental Figure 5.

### [^18^F]FAZA production, imaging, and analysis.

Twenty-four hours after completing 1 cycle of therapy (described in *Flow cytometry*), mice were prepared for [^18^F]FAZA assessment as previously described (58).

### RNA extraction, sequencing, and analysis.

Twenty-four hours after completing 1 cycle of therapy (described in *Flow cytometry*), RNA was extracted from dissociated tumors using RNeasy Mini Kit (QIAGEN, 74104) and sent to Avera for sequencing using 200 bp paired-end runs. Fastq files were aligned to the murine reference genome using kallisto (v0.46.1). R and Bioconductor packages limma (v3.48.3) and edgeR (v3.34.0) were used to identify differentially expressed genes with FDR < 0.01 and |fold-change| ≥ 1.5. Bioconductor package GSEAbase (v1.54.0) was used to conduct GSEA. For analysis of overlap between gene sets, fold-change was set to ± 1.5 and FDR ≤ 0.25. TCGA PAAD gene expression profiles were acquired using the University of California, Santa Cruz, Xena database (https://xena.ucsc.edu/). The TAN signature was described previously (33).

### FACS and qPCR.

Implanted MT4-mCherry cells were treated and resected as described in Figure 6C before processing as described under *Flow cytometry*. Whole-tumor single-cell suspensions were sorted using a BD FACSAria III. Reverse transcription and qPCR analysis were described previously; briefly, 50 ng of RNA was used as template for cDNA synthesis using SSIV (Invitrogen, 18091200). Samples were run in triplicate on a ViiA 7 Real-Time PCR System, and relative expression of *Ccl9* (Mm00441260_m1) was normalized to *Hprt* (Mm03024075_m1) and calculated using the 2^−ΔΔCT^ method (57).

### Bone marrow adoptive transfer.

Orthotopic pancreatic tumors were established as described above. Fourteen days after implantation and after the third dose of TH-302, naive CD11b^+^ myeloid cells were isolated from the bone marrow of naive mice using CD11b MicroBeads (Miltenyi Biotec, 130-049-601) and stained using CFSE (Invitrogen, C34554) per manufacturer’s instructions. A total of 3 × 10^6^ labeled myeloid cells in 100 μL of PBS were delivered by i.v. injection. At 60 hours postinjection, pancreatic tumors were resected and processed as described above, and immune infiltrate was analyzed for the frequency of CD11b^+^Ly6C^mid/hi^Ly6G^+^CFSE^+^ cells. The relative frequency of CFSE-labeled cells was calculated using the frequency of CFSE^+^ cells from the parent gate CD11b^+^Ly6G^+^. Frequency percentages were normalized to the untreated condition to derive the relative frequency of CFSE^+^ cells that migrated in the presence of combination-treated versus untreated tumor lysate.

### Ly6G depletion.

Anti-Ly6G antibody (clone 1A8, 25 μg/mouse for the first week, then 50 μg/mouse every day) and anti-Rat Kappa Immunoglobulin Light Chain (clone MAR18.5, 50 μg/mouse every other day) were delivered by IP injection following the scheme presented in Figure 5 (29). This strategy has demonstrated successful depletion of Ly6G^+^ cells in tumor tissue and peripheral tissue (59–61). On day 16, depletion of Ly6G^+^ cells was assessed in the peripheral blood versus tumor tissue, as mice that received depletion antibodies were enrolled in a survival study. To prevent false-negative staining due to epitope masking from the 1A8 clone, a CD11b^+^Ly6C^mid^-based gating strategy was used to approximate the percentage of G-MDSCs in peripheral blood (29, 62). Peripheral blood was collected via retro-orbital bleeding, and red blood cells were lysed (MilliporeSigma, R7757-100ML) before samples were fixed overnight at 4°C using the Foxp3/Transcription Factor Fixation/Permeabilization kit. Samples were prepared for flow cytometry as described in *Flow cytometry*.

### Preparation of tumor protein extracts.

Twenty-four hours after completing 1 cycle of therapy (described in *Flow cytometry*), 10-day implanted pancreatic tumors were resected and processed as recommended by Proteome Profiler Mouse XL Cytokine Array (R&D Systems ARY028) and Mouse Chemokine Array Kits (R&D Systems, ARY020). Briefly, tumors were homogenized using 5 mm stainless steel beads (QIAGEN, 69989) in a TissueLyser II (QIAGEN, 85300) at 25 Hz for 2 minutes. Triton X-100 was added to a final concentration of 1% before samples were frozen overnight, thawed, and then centrifuged at 10,000*g* for 5 minutes at 4°C to remove cell debris. Tumor lysate protein concentrations were quantified using Pierce BCA Protein Assay Kit (Thermo Fisher Scientific, 23225). A total of 200 μg of protein pooled from 2 or 3 tumors per group was incubated on provided nitrocellulose membranes. For the Mouse CCL9/10/MIP-1 gamma DuoSet ELISA (R&D Systems, DY463), lysates from bulk tumor samples were diluted 1:100 prior to following manufacturer’s instructions.

### Transwell assay.

Lysate derived from bulk tumor samples (described in *Preparation of tumor protein extracts*) was plated on the bottom of a 12-well Corning Costar Transwell, diameter 6.5 mm, pore size 8.0 μm, with 1 mL of complete RPMI. A total of 1 × 10^6^ naive myeloid cells derived from bone marrow were seeded in the polycarbonate membrane insert, and migrated cells were collected 48 hours later for flow cytometry analysis using CountBright Absolute Counting Beads (Invitrogen, C36950) (17). For CCR1 antagonist treatment, isolated cells were stained with CFSE, then pretreated with 20 μM BX471 or vehicle (DMSO) in PBS for 30 minutes before being seeded in the Transwell insert.

### Statistics.

GraphPad Prism v.9.0 was used for statistical analysis and plotting data. Kaplan-Meier survival curves were compared using log-rank Mantel-Cox test. Intergroup comparisons were assessed using Student’s 2-tailed *t* tests or 1- or 2-way ANOVA. Significance is considered at *P* < 0.05.

### Study approval.

All animal experiments were performed in compliance with the protocol (00001378-RN01/RN02) approved by the Institutional Animal Care and Use Committee at The University of Texas MD Anderson Cancer Center.

### Data availability.

RNA-Seq data are available in National Center for Biotechnology Information Gene Expression Omnibus under accession number GSE220217. Supporting Data Values for graphs are provided in the supplemental materials.

## Author contributions

AL and MAC conceived the study; AL, STG, and CRA developed methodology; AL, STG, AB, and FP investigated; AL and STG performed formal analysis; STG, DSH, MAC, and DPW provided resources, financial support, or coordination of research activity; MAC and DPW supervised; AL wrote the manuscript; and AL and MAC reviewed and edited the manuscript.

## Supplementary Material

Supplemental data

Supporting data values

## Version 1 11/21/2023

In-Press Preview

## Version 2 01/09/2024

Electronic publication
