# Supplementary material for: Hypoxia-activated prodrug and antiangiogenic therapies cooperatively treat pancreatic cancer but elicit immunosuppressive G-MDSC infiltration
Source: JCI Insight. 2024 Jan 9;9(1):e169150. doi: 10.1172/jci.insight.169150 (PMC10906452; doi:10.1172/jci.insight.169150)
Supplement: Supplemental data [file jciinsight-9-169150-s040.pdf]

Supplementary Figures and Legends

JCI Insight (Revisions 2023) – Arthur Liu *et al.*

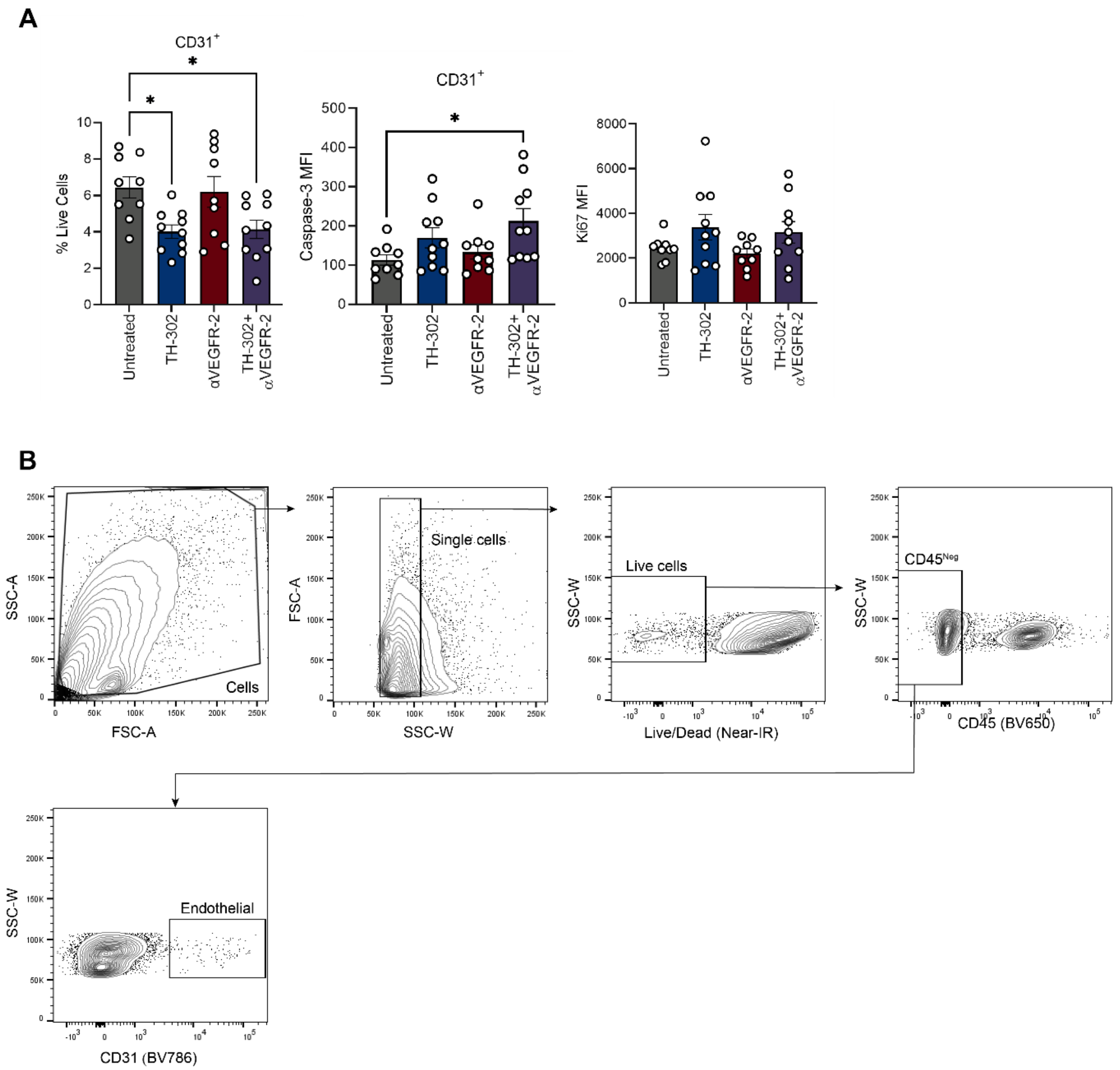

Supplementary Figure 1. Combination TH-302 and αVEGFR-2 results in endothelial cell pruning. 10-day established MT4-LA tumors were treated for five consecutive days of TH-302 and two doses of αVEGFR-2 prior to resection and processing for flow cytometry analysis. For endothelial cell analysis, the pellet fraction from the Ficoll density gradient separation was collected. (**A**) $CD31^+$ cells were assessed for their total frequency and expression of Active Caspase-3 and Ki67 (*n* = 9-10 mice per group). (**B**) Representative gating strategy for $CD31^+$ endothelial cells. One-way ANOVA followed by Tukey's correction for multiple comparisons. **P adj* < 0.05, ***P adj* < 0.01, ****P adj* < 0.001, *****P adj* < 0.0001.

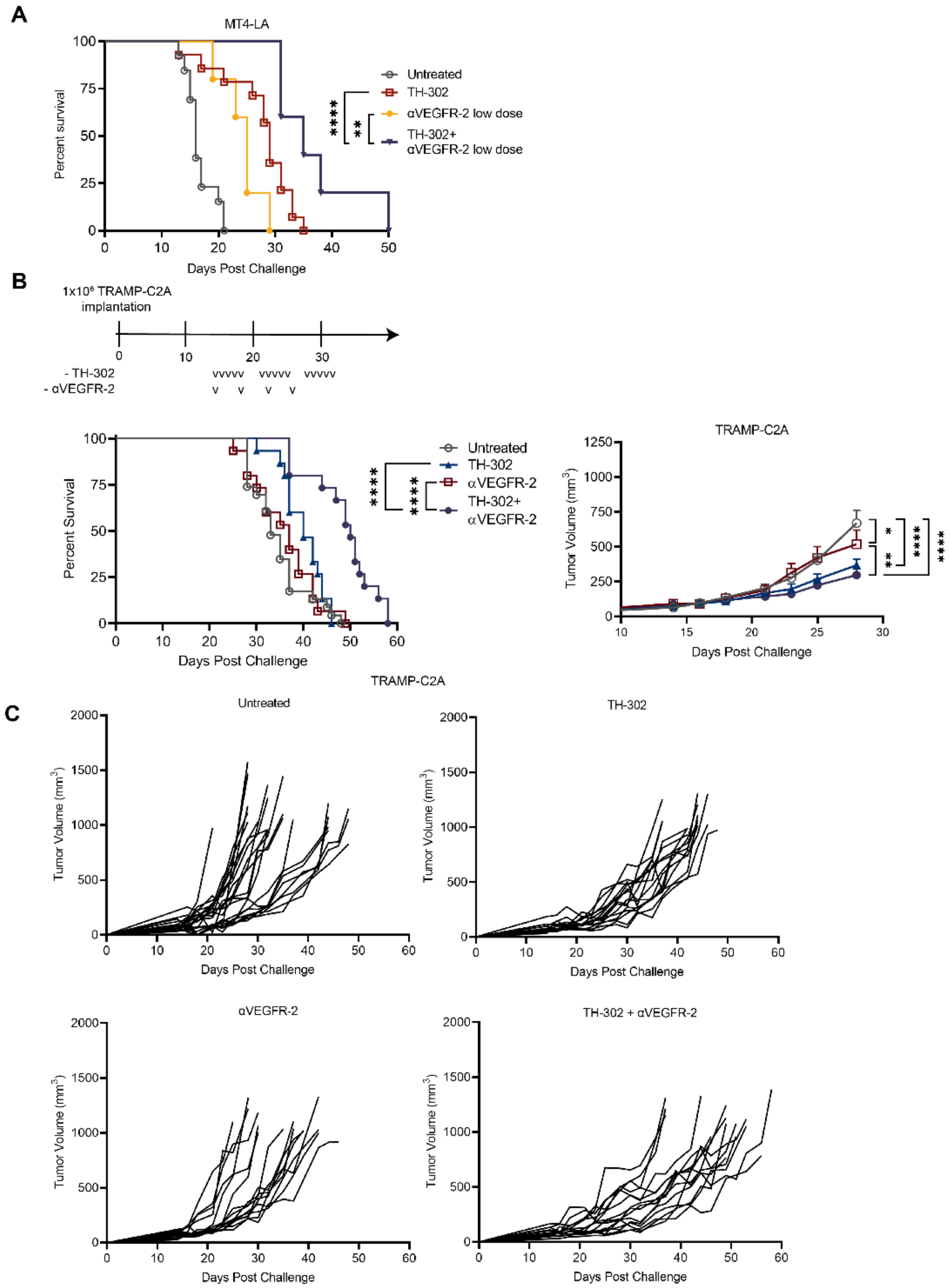

Supplementary Figure 2. Low dose αVEGFR-2 treatment of orthotopic pancreatic tumors and survival of mice challenged with TRAMP-C2A tumors. (**A**) Survival of mice treated with 10 mg/kg αVEGFR-2 (*n* = 5-10 mice per group). Untreated and TH-302 treated mice data is first presented in Figure 2. (**B**) Mice bearing 14-day pre-implanted subcutaneous TRAMP-C2A prostate tumors were treated as described and monitored for survival and tumor growth (*n* = 15-23 mice per group). (**C**) Individual tumor growth curves of TRAMP-C2A shown in (**B**). Log-rank (Mantel-Cox) test for survival and two-way ANOVA followed by Tukey's correction for multiple comparisons for tumor growth. **P adj* < 0.05, ***P adj* < 0.01, ****P adj* < 0.001, *****P adj* < 0.0001; data are mean ± SEM.

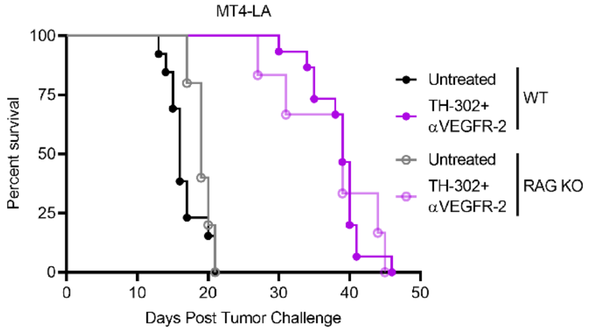

Supplementary Figure 3. Combination TH-302 and VEGFR2 blockade is not dependent on the adaptive immune response. 10 day established MT4-LA tumors in wild type or *Rag*-knockout mice were treated with TH-302 and VEGFR-2 as described in Figure 2 (n = 5-15 per group. Wild type mouse data is first presented in Figure 2. Log-rank (Mantel-Cox) test for survival. **P adj* < 0.05, ***P adj* < 0.01, ****P adj* < 0.001, *****P adj* < 0.0001; data are mean ± SEM.

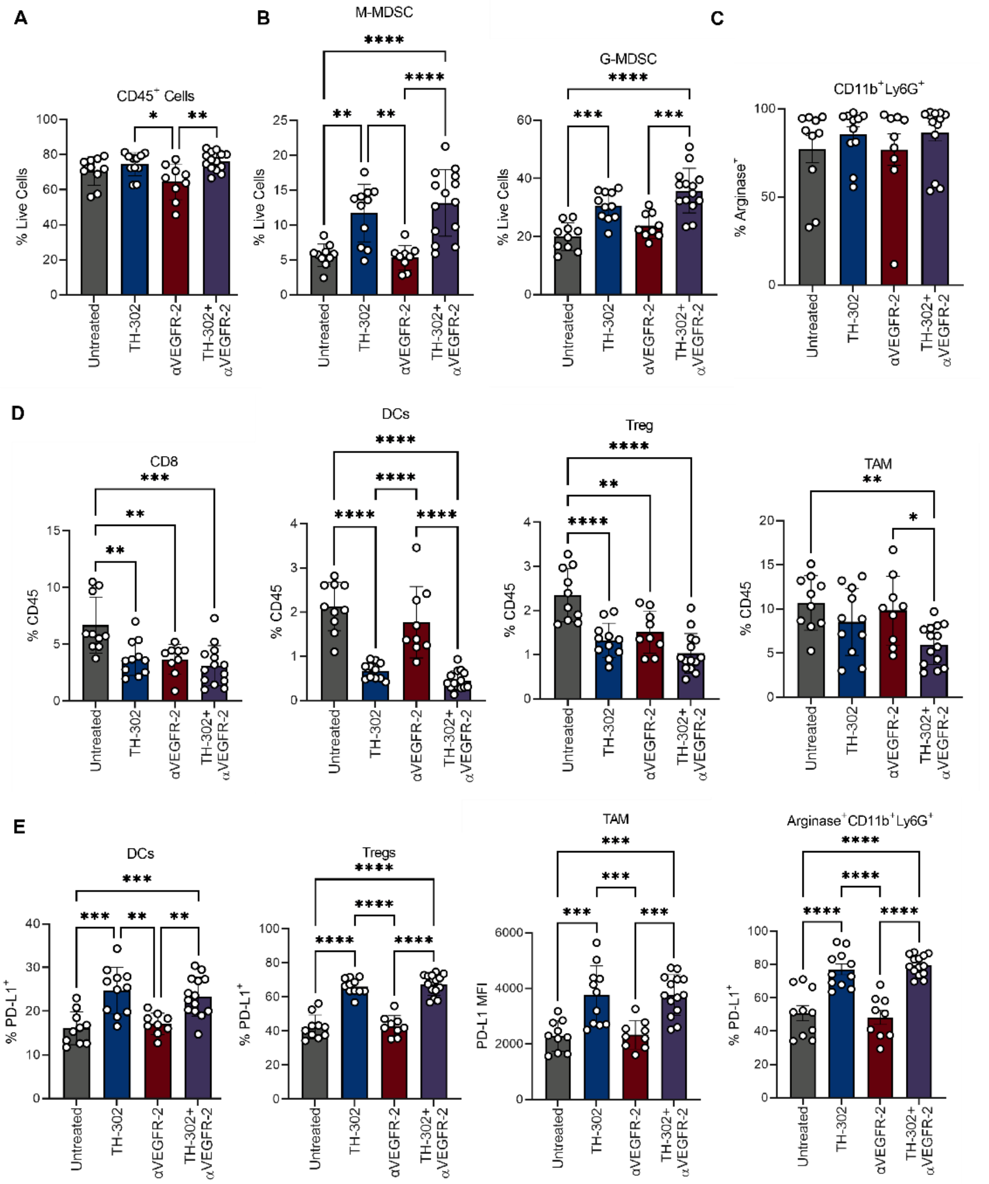

Supplementary Figure 4. Effect of TH-302 and VEGFR-2 blockade on tumor infiltrating immune cells. 10-day established MT4-LA tumors were treated for five consecutive days of TH-302 and two doses of VEGFR-2 blockade prior to resection for flow cytometry analysis. (**A**) Percent of live, CD45^{+} cells. (**B**) Percentage of live, M-MDSC and G-MDSC. (**C**) Proportion of Ly6G positive cells that express Arginase 1. (**D**) Proportion of CD8 T cells, dendritic cells (DCs), CD4 T regulatory cells (Treg), and tumor associated macrophages (TAMs). (**E**) Frequency of PD-L1 expressing DCs, Tregs, and TAMs (**A**-**E)** n = 10-14 per group. One-way ANOVA followed by Tukey's correction for multiple comparisons. **P adj* < 0.05, ***P adj* < 0.01, ****P adj* < 0.001, *****P adj* < 0.0001; data are mean ± SEM.

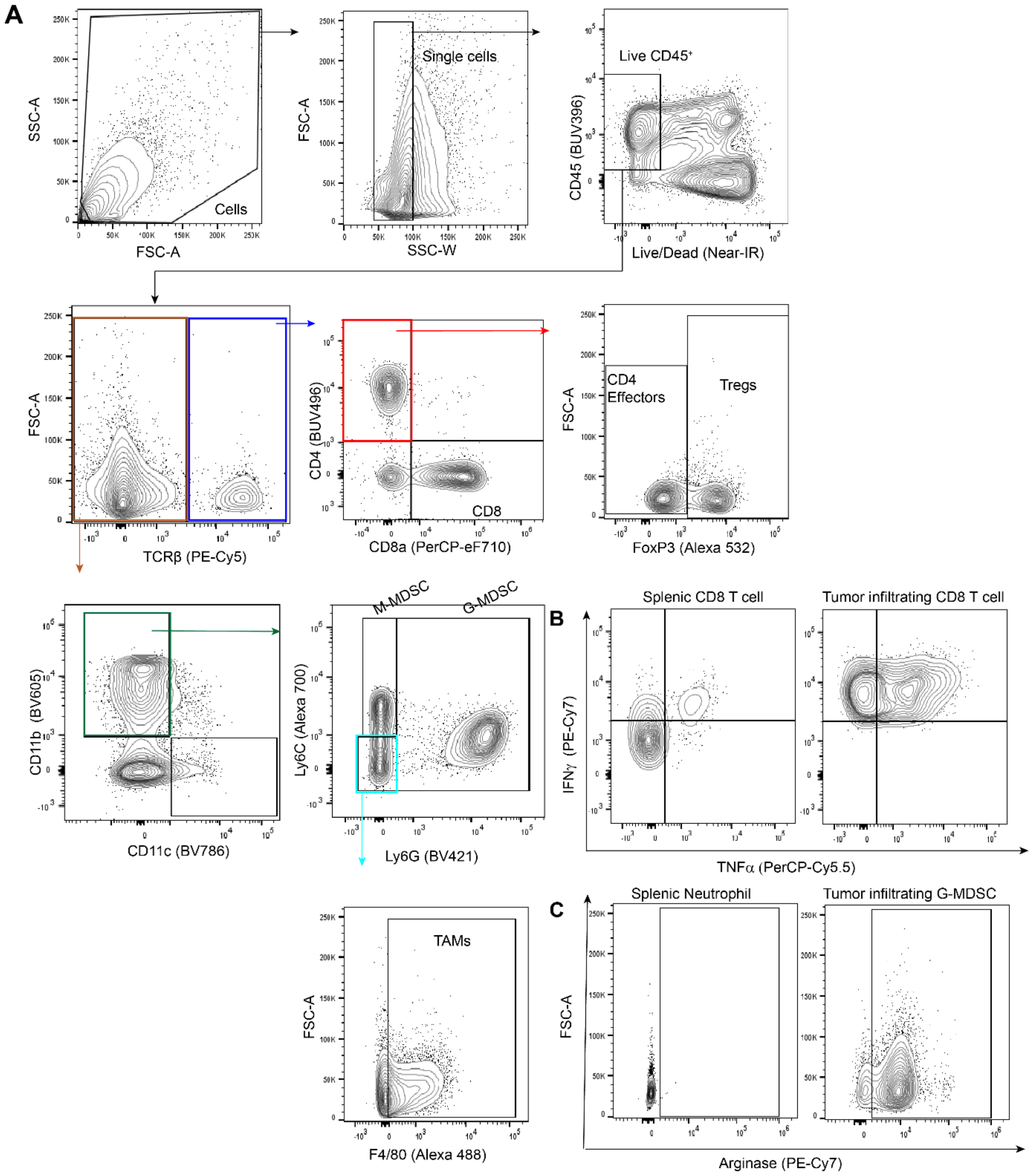

Supplementary Figure 5. Tumor infiltrating immune cell gating schema. (**A**) Representative gating for live, tumor infiltrating immune cells and representative gating for (**B**) dual expressing IFNγ and TNFα T cells and (**C**) arginase gating on Ly6G$^+$ cells.

A

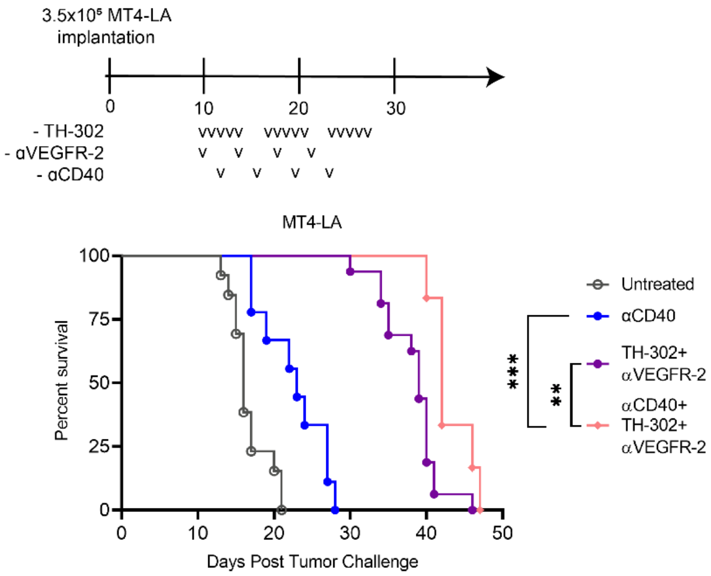

B

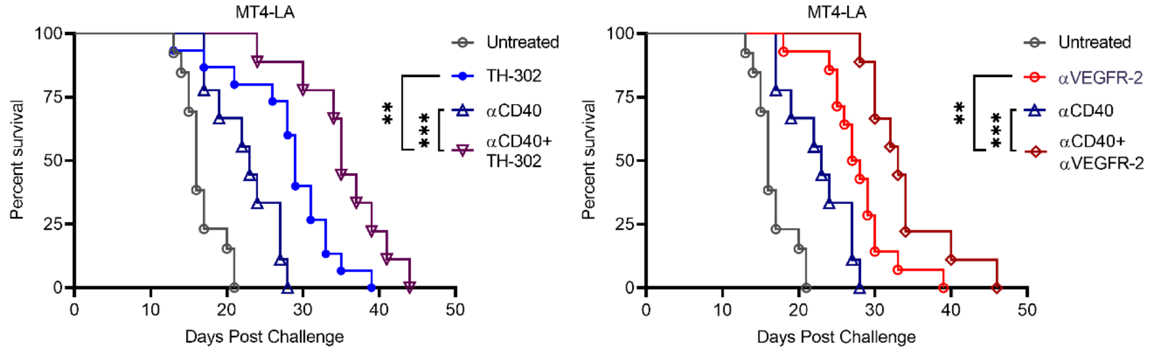

Supplementary Figure 6. αCD40 provides an additive benefit when combined with either TH-302 or αVEGFR-2. (**A**) αCD40 used in combination with TH-302 and αVEGFR-2 or (**B**) with each monotherapy (n = 9-15 mice per group). αCD40 survival curves presented in (**B**) are the same but presented in two separate graphs for clarity. Untreated and TH-302 treated mice are first presented in Figure 2. Log-rank (Mantel-Cox) test. *P *adj* < 0.05, **P *adj* < 0.01, ***P *adj* < 0.001, ****P *adj* < 0.0001; data are mean ± SEM.

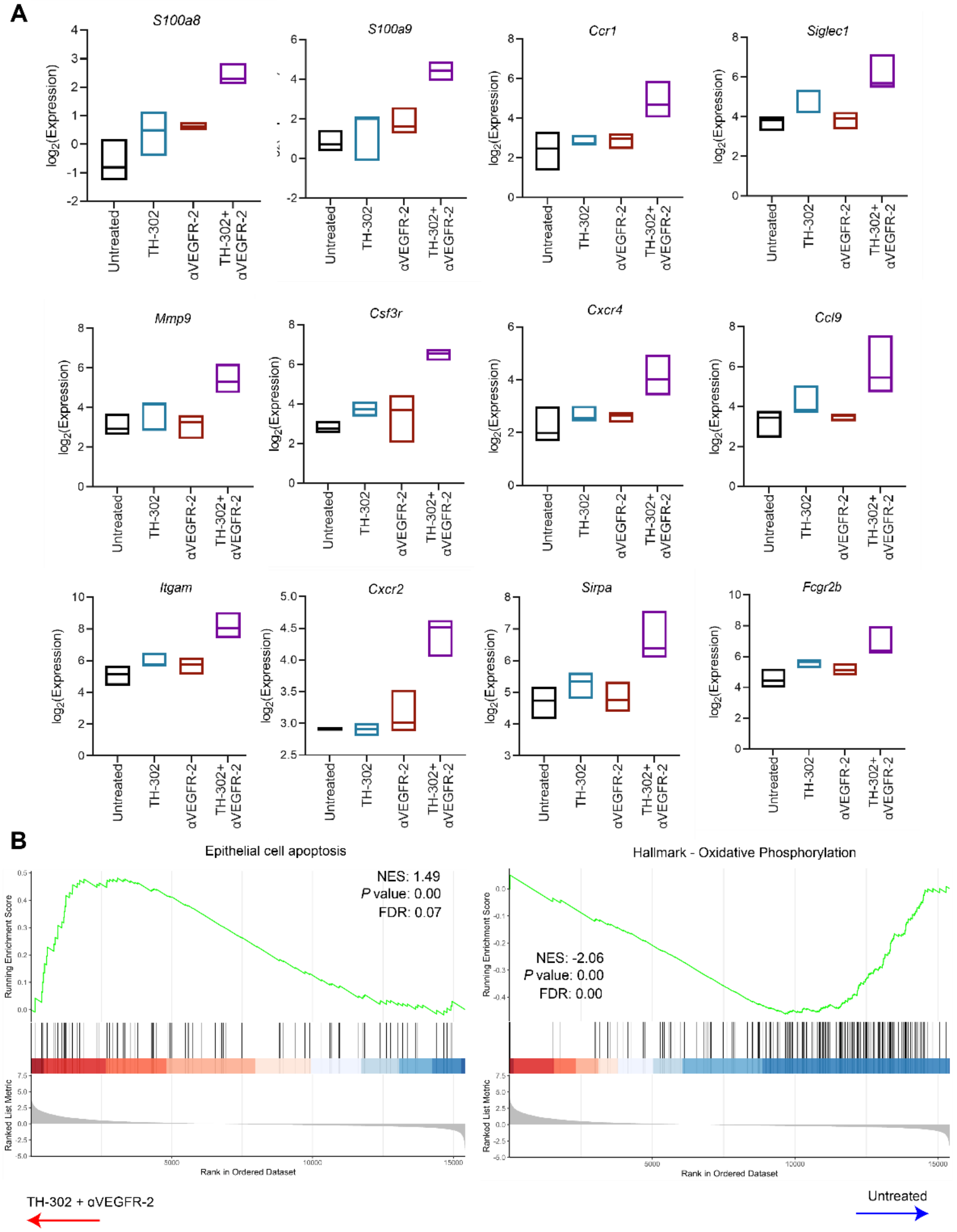

Supplementary Figure 7. Normalized expression levels of selected neutrophil-related genes expressed in treated tumors. (**A**) Genes enriched in combination treated tumors over untreated. (**B**) Leading edge analysis of epithelial cell apoptosis and hallmark oxidative phosphorylation (*n* = 3 mice per group). NES, normalized enrichment score. FDR, false discovery rate.

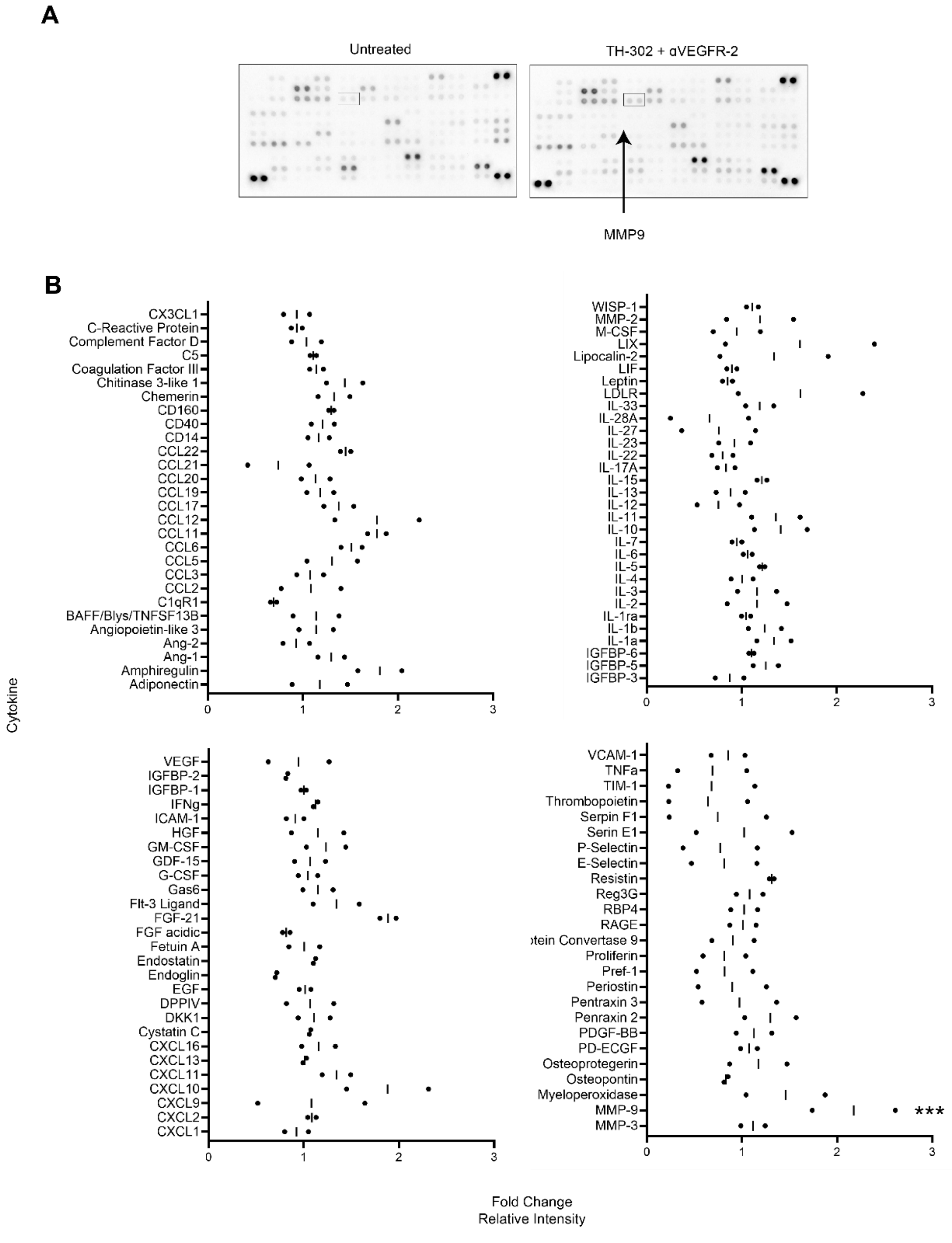

Supplementary Figure 8. MMP9 is enriched in combination TH-302 and αVEGFR-2 treated tumors. (**A**) Representative proteome Profiler Mouse XL Cytokine Array membranes and (**B**) associated quantification (n = 2-3 pooled tumors per group, 2 independent experiments). One-way ANOVA followed by Tukey's correction for multiple comparisons. *P adj < 0.05, **P adj < 0.01, ***P adj < 0.001, ****P adj < 0.0001; data are mean ± SEM.

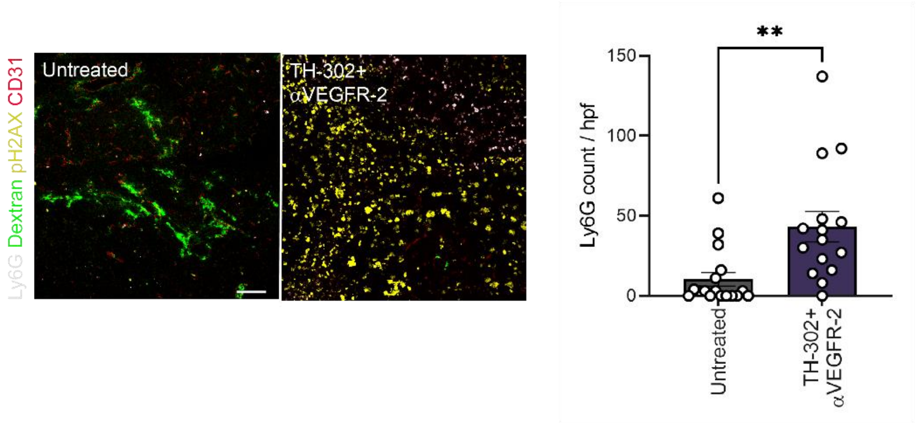

Supplementary Figure 9. Quantification of Ly6G positive cells in the tumor microenvironment. Left: Mice with untreated tumors or those that received one cycle of TH-302 and αVEGFR-2 treatment were injected with dextran prior to sacrifice. Tumors were resected, embedded in OCT for sectioning. Slides were stained with antibodies against Ly6G, pH2AX, and CD31. Right: Quantification of Ly6G counts per high power field. Unpaired two-tailed Student's *t* test.**P adj* < 0.05, ***P adj* < 0.01, ****P adj* < 0.001, *****P adj* < 0.0001; data are mean ± SEM.

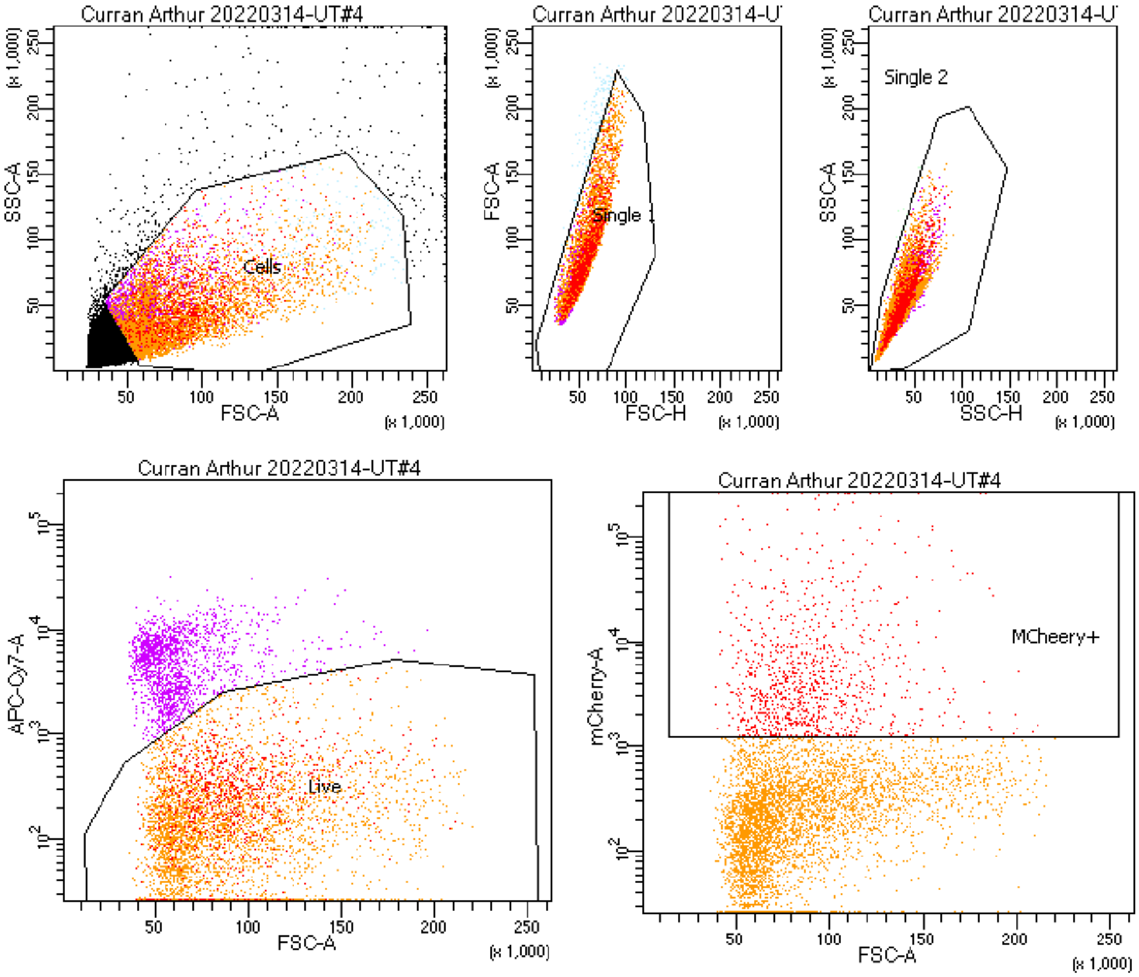

Tube: UT#4

Population	#Events	%Parent	%Total
All Events	30,000	####	100.0
Cells	7,685	25.6	25.6
Single 1	7,514	97.8	25.0
Single 2	7,503	99.9	25.0
Live	5,727	76.3	19.1
MCheery+	948	16.6	3.2

Supplementary Figure 10. Gating scheme for sorting mCherry positive pancreatic tumor cells for RNA extraction. Implanted pancreatic tumors were treated and processed as described in Figure 6 and Methods section before fluorescence-activated cell sorting on a BD FACSAria

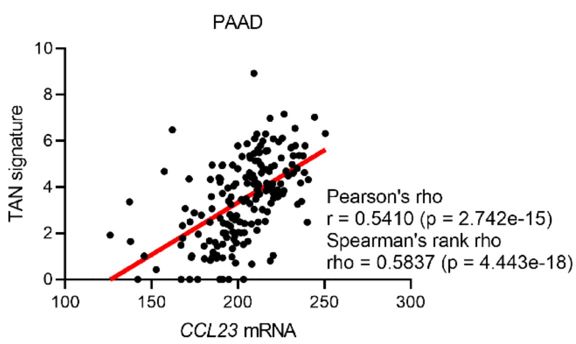

Supplementary Figure 11. Pearson's rho correlation between a TAN gene signature and *CCL23* or *CCR1* transcript in The Cancer Genome Atlas pancreatic adenocarcinoma (PAAD) dataset (*n* = 183).

Supplementary Table 1. List of antibodies and reagents used for flow cytometry and immunofluorescence

REAGENT or RESOURCE	SOURCE	IDENTIFIER
**Flow cytometry**		
CD45.2 – clone 104	BD Biosciences	564616
CD4 – clone GK1.5	BD Biosciences	612952
PD-1 – clone RMP1-30	BD Biosciences	748267
LAG-3 – clone C9B7W	BioLegend	125227
CD11c – clone HL3	BD Biosciences	563735
Ly6G – clone 1A8	BD Biosciences	562737
Ki67 – clone B56	BD Biosciences	563462
CD11b – clone M1/70	BD Biosciences	563015
IA/IE – clone M5/114	BD Biosciences	563415
F4/80 – clone BM8	Invitrogen	MF48020
CD8a – clone SK1	Invitrogen	46-0087-42
FoxP3 – clone FJK-16s	Invitrogen	58-5773-82
Tox – clone REA473	Miltenyi Biotec	130-120-716
TCRbeta – clone H57-597	Invitrogen	15-5961-82
Arginase – clone A1exF5	Invitrogen	25-3697-82
Active Caspase-3 – clone C92-605	BD Biosciences	564094
Ly6C – clone AL-21	BD Biosciences	561237
Near-IR Live/Dead	Invitrogen	L34975
CD31 – clone 390	BioLegend	102435
PD-L1 – clone MIH5	BD Biosciences	563369
CCR1 – clone S15040E	BioLegend	152507
CXCR4 – clone 2B11	BD Biosciences	551966
CXCR2 – clone V48-2310	BD Biosciences	747812
CSF3R – clone N/A	R&D Systems	FAB6039G
IFNγ - clone XMG1.2	Invitrogen	25-7311-41
Sirpα - clone P84	Invitrogen	25-1721-82
TNFα - clone MP6-XT22	BioLegend	506321
**Immunofluorescence**		
Anti-pimonidazole – clone 4.3.11.3	Hypoxyprobe	HP6-1000Kit
DAPI – clone N/A	Invitrogen	D1306
CD31 – clone 390	BD Biosciences	563608
Ki67 – clone D3B5	Cell Signaling Technology	9129
NG2 – clone N/A	Millipore	AB5320
pH2AX (Ser139) – clone 20E3	Cell Signaling Technology	9718
Ly6G – clone 1A8	BD Biosciences	562737
Donkey anti-Rabbit IgG (H+L) Highly Cross-Adsorbed Secondary Antibody, Alexa Fluor™ 488	Thermo Fisher Scientific	A-21206

Goat anti-Rat IgG (H+L) Highly Cross-Adsorbed Secondary Antibody, Alexa Fluor Plus 405	Thermo Fisher Scientific	A48261
Cleaved Caspase-3 (Asp175) Antibody #9661	Cell Signaling Technology	9661S

sSupplementary Table 2. List of critical resources and reagents

REAGENT or RESOURCE	SOURCE	IDENTIFIER
Fetal Bovine Serum	Gibco	26140079
DMEM	Gibco	11995065
Penicillin-Streptomycin	Thermo Fisher Scientific	15140122
Polybrene	Sigma-Aldrich	#TR-1003-G
Corning® Matrigel® Growth Factor Reduced (GFR) Basement Membrane Matrix, Phenol Red-free, LDEV-free	Corning	356231
Insulin solution from bovine pancreas	Sigma-Aldrich	I0516-5ML
Standard Insulin Syringe with Needle EasyTouch™ 0.5 mL 1/2 Inch 29 Gauge NonSafety Thin Wall	MHC Medical	829555
Phosphate Buffered Saline, pH 7.2	Gibco	20012050
InVivoMAb anti-mouse VEGFR-2 Antibody	Bio X Cell	BE0060
Anti-Mouse CTLA-4 (Clone 9H10) – Purified in vivo PLATINUM™ Functional Grade	Leinco Technologies	C2841
Anti-Mouse CD279 (PD-1) (Clone RMP1-14) – Purified in vivo PLATINUM™ Functional Grade	Leinco Technologies	P372
InVivoMAb anti-mouse Ly6G	Bio X Cell	BE0075-1
InVivoMAb anti-rat Kappa Immunoglobulin Light Chain	Bio X Cell	BE0122
InVivoMAb anti-mouse CD40	Bio X Cell	BE0016-2
Foxp3 / Transcription Factor Fixation/Permeabilization	Invitrogen	00-5521-00
Anti-Mouse CD32/CD16 – Purified in vivo PLATINUM™ Functional Grade	Leinco Technologies	C681
Fisherbrand™ Color-Coded Capillary Tubes	Fisher Scientific	22-260950
BD Vacutainer™ Plastic Blood Collection Tubes with Sodium Heparin: Hemogard™	Fisher Scientific	23-021-017
Red Blood Cell Lysing Buffer Hybri-Max	Sigma-Aldrich	R7757-100ML
Tris Buffered Saline with Tween® 20	Cell Signaling Technology	9997
Bovine Serum Albumin	Sigma-Aldrich	A2934-100G
Hypoxyprobe Plus Kit	Hypoxyprobe	HP2-200Kit

Fluorescein isothiocyanate–dextran average mol wt 2,000,000	Sigma-Aldrich	FD2000S
Histopaque-1119	Sigma-Aldrich	11191-6X100ML
CD8 T cell isolation Kit	Miltenyi-Biotec	130-104-075
eBioscience™ Cell Stimulation Cocktail (plus protein transport inhibitors) (500X)	Thermo Fisher Scientific	00-4975-93
RNeasy Mini Kit	Qiagen	74104
SuperScript™ IV First-Strand Synthesis System	Thermo Fisher Scientific	18091200
CD11b MicroBeads, human and mouse - lyophilized	Miltenyi-Biotec	130-097-142
CellTrace™ CFSE Cell Proliferation Kit, for flow cytometry	Thermo Fisher Scientific	C34554
Proteome Profiler Mouse XL Cytokine Array	R&D Systems	ARY028
Proteome Profiler Mouse Chemokine Array Kit	R&D Systems	ARY020
Stainless Steel Beads, 5 mm	Qiagen	69989
Tissue Lyser II	Qiagen	85300
Pierce™ BCA Protein Assay Kit	Thermo Fisher Scientific	23225
12-well Corning® Costar® Transwell®, diam. 6.5 mm, pore size 8.0 µm	Corning	3422
CountBright™ Absolute Counting Beads	Thermo Fisher Scientific	C36950
BX471	MedChemExpress	HY-12080
Mouse CCL9/10/MIP-1 gamma DuoSet ELISA	Biotechne | R&D Systems	DY463
